# Retinoic Acid Accelerates the Specification of Enteric Neural Progenitors from *In-Vitro*-Derived Neural Crest

**DOI:** 10.1016/j.stemcr.2020.07.024

**Published:** 2020-08-27

**Authors:** Thomas J.R. Frith, Antigoni Gogolou, James O.S. Hackland, Zoe A. Hewitt, Harry D. Moore, Ivana Barbaric, Nikhil Thapar, Alan J. Burns, Peter W. Andrews, Anestis Tsakiridis, Conor J. McCann

**Affiliations:** 1 University of Sheffield, Department of Biomedical Science, Sheffield, UK; 2 The Center for Stem Cell Biology, Memorial Sloan Kettering Cancer Center, New York, USA; 3 Stem Cells and Regenerative Medicine, UCL Great Ormond Street Institute of Child Health, London, UK; 4 Neurogastroenterology and Motility Unit, Great Ormond Street Hospital, London, UK; 5 Department of Gastroenterology, Hepatology and Liver Transplant, Queensland Children's Hospital, Brisbane, Australia; 6 Prince Abdullah Ben Khalid Celiac Research Chair, College of Medicine, King Saud University, Riyadh, KSA; 7 Department of Clinical Genetics, Erasmus University Medical Center, Rotterdam, The Netherlands

**Keywords:** pluripotent stem cells, embryonic stem cells, human, neural crest, enteric nervous system, cell transplantation, Hirschsprung disease, retinoic acid

## Abstract

The enteric nervous system (ENS) is derived primarily from the vagal neural crest, a migratory multipotent cell population emerging from the dorsal neural tube between somites 1 and 7. Defects in the development and function of the ENS cause a range of enteric neuropathies, including Hirschsprung disease. Little is known about the signals that specify early ENS progenitors, limiting progress in the generation of enteric neurons from human pluripotent stem cells (hPSCs) to provide tools for disease modeling and regenerative medicine for enteric neuropathies. We describe the efficient and accelerated generation of ENS progenitors from hPSCs, revealing that retinoic acid is critical for the acquisition of vagal axial identity and early ENS progenitor specification. These ENS progenitors generate enteric neurons *in vitro* and, following *in vivo* transplantation, achieved long-term colonization of the ENS in adult mice. Thus, hPSC-derived ENS progenitors may provide the basis for cell therapy for defects in the ENS.

## Highlights

• Retinoic acid alters the axial identity of hPSC-derived neural crest cells
• ENS progenitor markers are upregulated in response to RA
• ENS progenitors are capable of generating enteric neurons *in vitro*
• hPSC ENS progenitors colonize the ENS of mice following long-term transplantation

## Teaser

In this article, Frith and colleagues show that retinoic acid (RA) signaling alters the axial identity of hPSC-derived neural crest cells in a time- and dose-dependent manner. They utilized this to derive enteric nervous system (ENS) progenitors from hSPCs, which can differentiate to enteric neurons *in vitro* and colonize the ENS of adult mice following long-term transplantation.

## Introduction

The enteric nervous system (ENS) is the largest branch of the peripheral nervous system and consists of an extensive network of neurons and glia controlling critical intestinal functions, such as motility, fluid exchange, gastric acid/hormone secretion, and blood flow (reviewed in Sasselli et al., 2012). In amniotes, the ENS is derived predominantly from the vagal neural crest (NC), a multipotent cell type specified at the neural plate border between somites 1 and 7. The vagal NC contributes to structures in various other organs, such as the heart, thymus, and lungs (Hutchins et al., 2018; Le Douarin et al., 2004; Simkin et al., 2018). After delaminating from the dorsal neural tube, vagal NC cells migrate and enter the foregut where enteric neural progenitors colonize the developing gut in a rostro-caudal direction. Determinants of ENS progenitor migration, proliferation, and differentiation include the RET-GDNF (Durbec et al., 1996) and endothelin-3-EDNRB (Baynash et al., 1994; Hosoda et al., 1994) signaling pathways and the transcription factors *SOX10*, *PHOX2B*, and *ASCL1* (Bondurand et al., 2006; Elworthy et al., 2005; Memic et al., 2016). However, the signals that shape early ENS identity within vagal NC precursors remain less well defined.

Vagal NC cells express members of the HOX paralogous groups (PG) 3–5 (Diman et al., 2011; Fu et al., 2003; Kam and Lui, 2015) and are patterned mainly by the action of somite-derived retinoic acid (RA) signaling, which acts by “posteriorizing” cranial HOX^−^ NC progenitors (Frith et al., 2018; Ishikawa and Ito, 2009; Stuhlmiller and García-Castro, 2012). *In vivo* studies implicate RA in the specification of downstream vagal NC derivatives (El Robrini et al., 2016; Niederreither et al., 2001, 2003), particularly the ENS where RA signaling components control progenitor migration and proliferation (Niederreither et al., 2003; Uribe et al., 2018).

hPSCs offer an attractive approach for dissecting early cell fate decisions. To date, few studies have described the generation of ENS progenitors and neurons from PSCs indicating that these cell populations can be used to model and treat enteric neuropathies, such as Hirschsprung disease (HSCR) (Fattahi et al., 2016; Lai et al., 2017; Li et al., 2016; Workman et al., 2016). These protocols rely on transforming growth factor β/BMP inhibition followed by exposure to WNT, BMP, and RA to form vagal NC, yielding ENS progenitors after 10–15 days in culture (Fattahi et al., 2016; Workman et al., 2016). However, the precise timing and concentration of RA signaling that control the positional identity of NC cells has not been clearly defined. Moreover, it is not yet clear whether RA imparts an early enteric neural identity in hPSC-derived vagal NC or acts solely as a positional specifier.

We previously described the efficient and robust production of NC cells from hPSCs (Frith et al., 2018; Hackland et al., 2017), which can acquire a vagal axial identity following exposure to RA (Frith et al., 2018). This method overcame variations in NC induction due to variable levels of endogenous BMP, typical of hPSC cultures, by using Top-down inhibition (Hackland et al., 2017) in which a saturating level of exogenous BMP supplements endogenous BMP and the signaling is precisely modulated by a BMP inhibitor. Using this system, we show that RA acts in a dose-dependent manner on pre-specified NC precursors to induce vagal *HOX* genes and direct the accelerated production of ENS progenitors that generate enteric neurons *in vitro* and colonize the ENS of adult mice following long-term transplantation. Our findings provide an efficient platform for *in vitro* modeling of human ENS development and disease, and development of cell therapy-based approaches for the treatment of such conditions.

## Results

### The Timing of RA Signaling Affects NC Specification *In Vitro*

**Figure 1:**
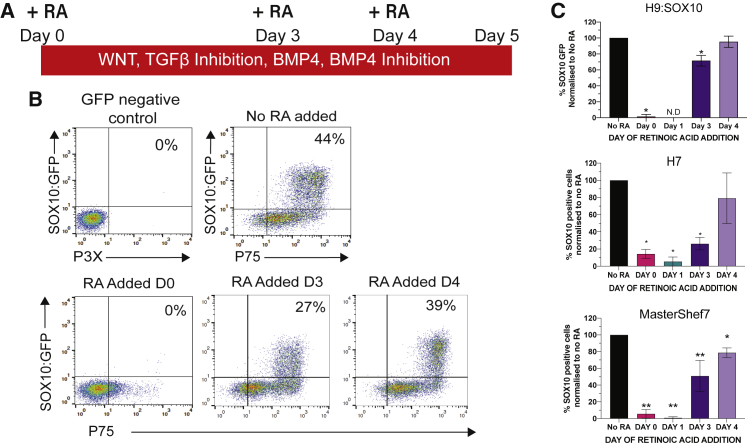
RA Affects NC Specification in a Time-Dependent Manner (A) Schematic of NC differentiation protocol and time points corresponding to addition of all-*trans* RA. (B) FACS plots showing *SOX10*:GFP and p75 expression at day 5 after RA addition at indicated time points during NC differentiation. (C) Percentage of cells expressing SOX10 in three hPSC lines following FACS or immunofluorescence. Graphs show percentage of SOX10+ cells normalized to cells not treated with RA. Bars = mean; error = SD. N = 4 independent differentiations for *SOX10*:GFP. N = 3 independent differentiations for H7 and MasterShef7. ^∗^p < 0.05, ^∗∗^p < 0.01; one-way ANOVA.

We previously showed that RA treatment of cranial NC precursors induces a vagal axial identity, defined by expression of HOX PG members 1–5 (Frith et al., 2018). To identify the developmental time window during which RA imparts a vagal identity without perturbing NC specification, we supplemented 1 μM all-*trans* RA at different stages of NC differentiation (Figure 1A). The NC markers p75 and *SOX10* were assessed by flow cytometry in a *SOX10*:GFP reporter hPSC line (Chambers et al., 2012). Adding RA at day 0 of differentiation did not yield any *SOX10*:GFP+/p75+ cells at day 5, while addition of RA at days 3 or 4 of differentiation saw similar levels of *SOX10*:GFP+/p75+ cells compared with untreated cells (Figures 1B and 1C). Immunostaining for SOX10 in two other hPSC lines (H7 and MasterShef7) confirmed the same temporal effect of RA on NC differentiation from hPSCs (Figures 1C and S1). While not affecting the efficiency of NC differentiation, RA did cause a variable reduction of the number of cells at day 6 of differentiation (Figure S1D), indicating low levels of RA toxicity. These data suggest that early RA signaling perturbs NC induction from hPSCs, while late addition of RA changes the axial identity of cells committed to NC fate.

### RA Induces Both Vagal and Enteric Neural Progenitor Identities in a Dose-Dependent Manner

**Figure 2:**
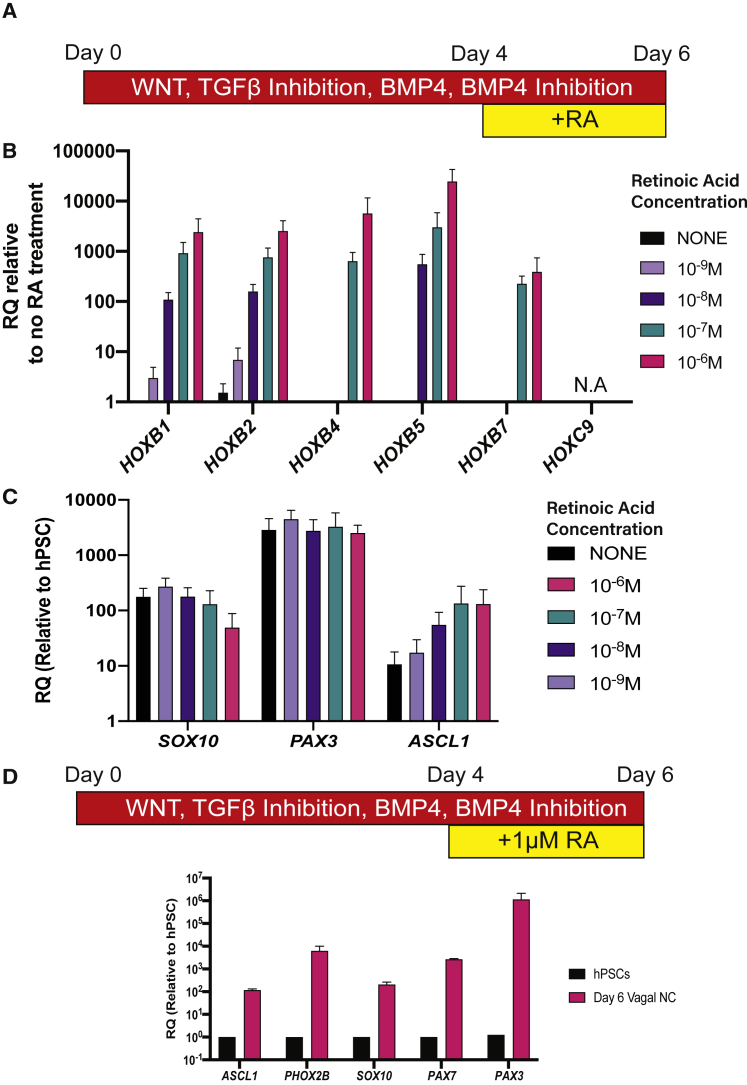
RA Induces a Vagal and ENS Progenitor Identity In a Dose-Dependent Fashion (A) Differentiation protocol to pattern hPSC-derived NC cells. (B and C) qPCR plots showing the induction of *HOX* genes (B) and NC/ENS markers (C) at day 6 relative to non-RA-treated *HOX negative* cells after exposure to different concentrations of RA. Bar = mean, error bars = SD, N = 3 independent differentiations of *SOX10*:GFP hPSCs. N.A., no amplification. (D) qPCR plots showing NC/enteric neural precursor markers in day 6 cells after 2 days exposure of 1 μM RA. Bar = mean, error bars = SD, N = 3 independent differentiations.

RA induces *HOX* gene expression in a dose-dependent manner *in vitro* (Okada et al., 2004; Simeone et al., 1990) and *in vivo* (Papalopulu et al., 1991). To examine how levels of RA signaling shape the axial identity of hPSC-derived NC cells, we treated day 4 HOX^−^ NC precursors with 10^−9^ M (1 nM) to 10^−6^ M (1 μM) RA and examined the expression of HOX and NC/ENS progenitor genes (Figure 2). *HOXB1* and *B2*, were induced by all concentrations of RA in a dose-dependent manner, while HOX genes marking vagal NC (*HOXB4*, *B5*, and *B7*) were only induced by higher RA concentrations (Figures 2B and S2), consistent with previous findings (Okada et al., 2004). No *HOXC9* expression was observed with any RA concentration, consistent with findings that truncal NC identity is mediated by WNT/FGF signaling (Abu-Bonsrah et al., 2018; Frith et al., 2018; Hackland et al., 2019; Lippmann et al., 2015).

Expression of the NC markers *SOX10*, *PAX7*, and *PAX3* was unaffected by the levels of RA (Figures 2C, 2D, and S2) in line with our previous observations (Figure 1). The highest concentrations of RA elicited higher expression of *ASCL1* and *PHOX2B* (Figure 2D) that mark peripheral nervous system precursors, including migrating ENS progenitors (Blaugrund et al., 1996; Elworthy et al., 2005; Lo et al., 1991). These results indicate that acquisition of a vagal axial identity and ENS progenitor specification in NC progenitors are tightly coupled events dependent on RA signaling.

### RA-Induced Vagal NC/ENS Progenitors Generate Putative Enteric Neurons *In Vitro*

**Figure 3:**
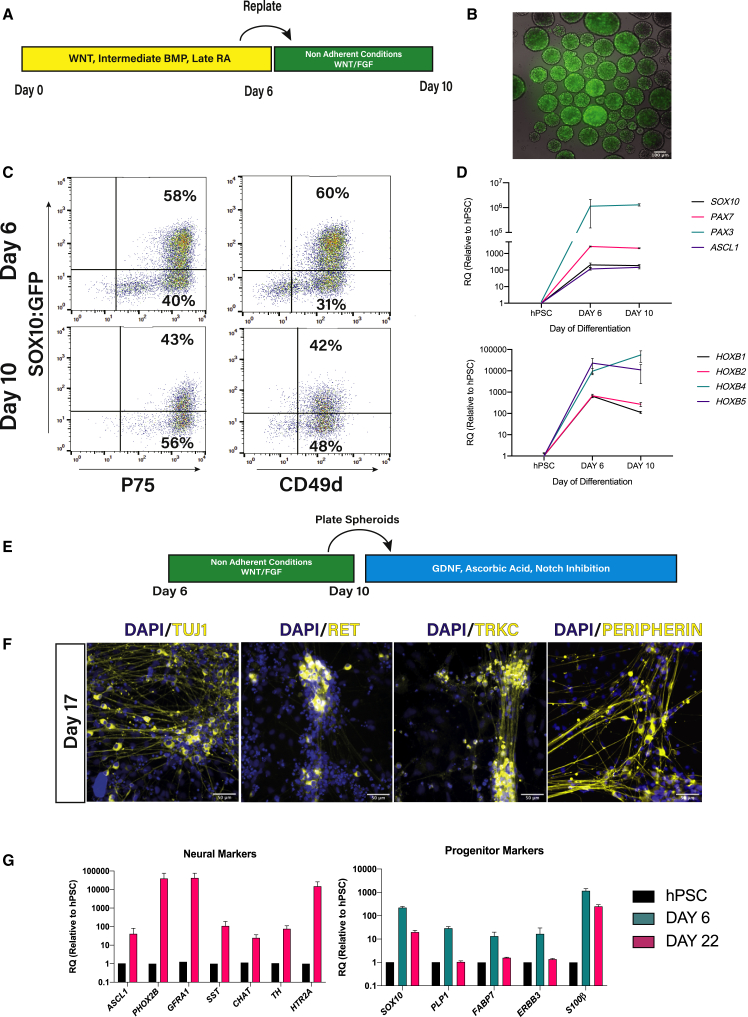
Day 6 Enteric Neural Precursors Can Generate Putative Enteric Neurons *In Vitro* (A) Schematic of non-adherent culture conditions. (B) Day 8 NC spheres containing *SOX10*:GFP+ cells. (C) FACS plots of *SOX10*:GFP and p75/CD49d expression in non-adherent conditions from day 6 to 10. (D) qPCR showing vagal NC/early ENS markers at days 6 and 10 of differentiation. Bars = mean; error = SD. N = 3 independent differentiations. (E) Enteric neuron differentiation conditions. (F) Immunofluorescence for enteric neuron markers at day 17 of differentiation. Scale bar = 50 μm. (G) qPCR analysis of enteric neuron and progenitor markers at day 22 of differentiation. Bars = mean; error = SD. N = 3 independent differentiations in SOX10:GFP hPSCs.

To test if day 6 vagal NC cells possess ENS progenitor potential, we tested their ability to form enteric neurons *in vitro*. Day 6 vagal NC cells were cultured in the presence of WNT/FGF in non-adherent conditions (Figure 3A), as described previously (Fattahi et al., 2016). Spheres retained *SOX10*:GFP expression, immunoreactivity of ENS precursor markers p75 and CD49d, and vagal NC and *HOX* gene expression (Figure 3D) after 4 days of culture (Figures 3B and 3C). At day 10, spheres were re-plated in conditions promoting enteric neuron differentiation (Figure 3E) (Fattahi et al., 2016; Okamura and Saga, 2008; Theocharatos et al., 2013). At day 17, we observed cells expressing the enteric neuronal markers TUJ1, RET, TRKC, and PERIPHERIN (Figure 3F). Similar results were obtained with two other hPSC lines (Figure S3). *ChAT*, *HTR2A*, *TH*, and *ASCL1* expression at day 22 further indicated the presence of early enteric neurons. The expression of glial/neuronal progenitor markers *SOX10* and *S100β* (Lasrado et al., 2017) were also detected in day 22 cultures, but were found to be reduced from day 6. *PLP1 ERBB3* and *FABP7* were expressed in day 6 ENS precursors, but switched off by day 22, consistent with the neurogenic effect of NOTCH inhibition (Figure 3G). These observations suggest that RA-induced NC cells can give rise to enteric neurons *in vitro*.

### RA-Induced Vagal NC/ENS Progenitors Colonize the Adult Mouse ENS *In Vivo*

**Figure 4:**
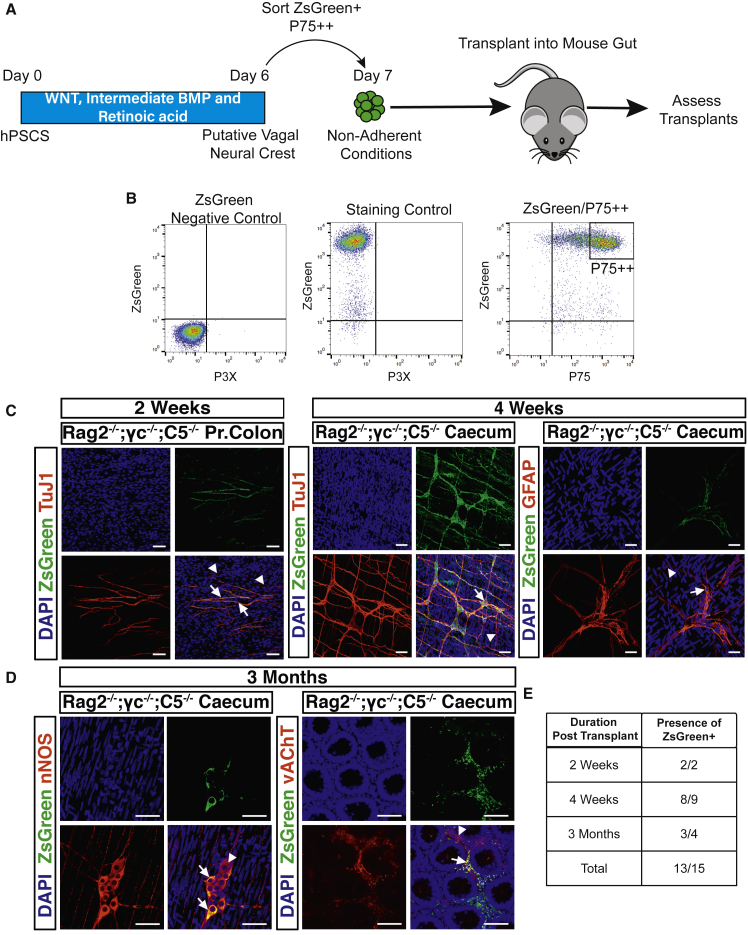
hPSC-Derived Enteric Neuronal Precursors Integrate into the Mouse ENS after Transplantation (A) Schematic of procedures for transplantation of hPSC-derived ENS progenitors. (B) Sorting strategy to isolate *in-vitro*-derived ZsGreen+/p75++-labeled putative ENS progenitors. (C) Whole-mount images of gut tissue corresponding to the indicated regions obtained at 2 and 4 weeks post-transplantation. Arrows show ZsGreen+ cells that are positive for TUJ1 among endogenous TUJ1+ neurons (arrowheads), and glial marker GFAP after immunostaining. Pr. Colon, proximal colon. Scale bar = 50 μm. (D) Images of differentiated hPSC-derived ENS progenitors into nNOS+ and vAChT+ neurons in the cecum of Rag2^−/−^;γc^−/−^;C5^−/−^ mice 3 months post-transplantation. Arrows show transplanted ZsGreen+ cells; arrowheads show endogenous enteric neurons. Scale bar = 50 μm. (E) Table showing the numbers of mice in which ZsGreen+ cells were identified over the total number of transplanted mice analyzed at indicated time points post-transplantation.

To assess the developmental potential of hPSC-derived vagal NC/ENS progenitors *in vivo*, we performed transplantation to the cecum of adult immunodeficient Rag2^−/−^;γc^−/−^;C5^−/−^ mice. To track transplanted cells, we used the PSC line SFCi55-ZsGr, which contains a constitutive ZsGreen reporter in the *AAVS* locus (Lopez-Yrigoyen et al., 2018). We generated spheres from day 6 ZsGreen+/p75++-sorted putative ENS progenitors. The next day, ZsGreen+/P75++ cells were transplanted to the serosal aspect of the cecum in adult (4–8 weeks) immunodeficient Rag2^−/−^;γc^−/-^;C5^−/−^ mice and analyzed for integration and differentiation at timed intervals (Figures 4A and 4B). Two weeks post-transplantation TUJ1+ ZsGreen+ cells (Figure 4C, left; arrowheads), were observed at the serosal aspect within the cecum and proximal colon (N = 2/2 mice). At 4 weeks post-transplantation ZsGreen+ cells were again observed on the serosal surface and within the tunica muscularis at the level of the myenteric plexus (N = 8/9 mice). Within the tunica muscularis, ZsGreen+ cells co-expressed TUJ1 both within myenteric ganglia-like structures and as intramuscular neurons (Figure 4C, right). We also detected ZsGreen+ cells at the level of the myenteric plexus, which were positive for the glial marker GFAP (Figure 4C, right). After 3 months, ZsGreen+ cells could be found across the gut wall both within individual myenteric ganglia (Figure 4D, left) and the submucosa, surrounding cryptal structures (N = 3/4 mice) (Figure 4D, right). Furthermore, there were ZsGreen+ cells that had differentiated into enteric neuronal subtypes expressing either neuronal nitric oxide synthase (nNOS) (Figure 4D, left) or vesicular acetylcholine transporter (vAChT) (Figure 4D, right). At this time, donor cell coverage averaged 2.8 ± 1.8 mm^2^ compared with 0.05 ± 0.02 mm^2^ after 2 weeks post-transplantation (N = 2 mice/time point). These results suggest that hPSC-derived ENS progenitors integrate within recipient gut and are maintained long-term, differentiating to multiple neuronal subtypes and glia.

## Discussion

We describe a differentiation system utilizing RA to drive the concomitant induction of both a vagal and an ENS progenitor identity from hPSCs. Top-down inhibition produces an intermediate level of BMP signaling that, in combination with WNT, robustly and efficiently generates NC after 5–6 days *in vitro* (Frith et al., 2018; Hackland et al., 2017), which go on to express vagal level HOX genes after RA signaling. This is quicker than previously published protocols that yield ENS progenitors after 10–15 days (Fattahi et al., 2016; Workman et al., 2016). We also report the induction of *ASCL1* and *PHOX2B* shortly after the addition of RA to NC precursors, which, combined with *SOX10* and p75 expression, is consistent with ENS progenitor identity (Figure 2). We previously showed that RA treatment of NC precursors also induces markers of cardiac and posterior cranial NC alongside ENS progenitor markers during vagal NC specification (Frith et al., 2018), suggesting that axial identity and cell fate are inter-linked.

Previous studies reveal a role for RA signaling in promoting ENS progenitor migration, proliferation, and differentiation (Niederreither et al., 2003; Simkin et al., 2013; Uribe et al., 2018). RA may control these processes through vagal HOX genes such as *HOXB3*, *HOXB5*, and TALE family co-factors, which regulate ENS development (Chan et al., 2005; Kam and Lui, 2015; Uribe and Bronner, 2015; Uribe et al., 2018) by inducing *Ret* (Zhu et al., 2014) and preventing apoptosis (Kam et al., 2014).

Our differentiation strategy rapidly and robustly yields a well-defined progenitor cell population that can generate enteric neurons *in vitro* (Figure 3). To test their potential as a cellular donor to treat enteric neuropathies, we transplanted our ENS progenitors into the gut of immunodeficient Rag2^−/−^;γc^−/−^;C5^−/−^ mice. This eliminated the requirement for chemical immunosuppression, allowing long-term study of donor cell survival and integration within a “normal” host ENS microenvironment. Crucially, we found that the hPSC-derived neurons were present within endogenous ENS ganglia of adult mice up to 3 months post-transplantation (N = 3/4), expressing the same markers (nNOS and vAChT) as host neuronal populations (Figure 4D). Transplanted human cells populated both the myenteric and submucosal plexuses of the gut, demonstrating extensive migration within the gut wall and formation of neuronal networks with close interactions with the intact host ENS (Figure 4).

Transplantation studies using postnatal human and murine endogenous enteric neural stem cells in mice demonstrated functional integration (Cooper et al., 2016, 2017; Stamp et al., 2017), and rescue of an enteric neuropathy (McCann et al., 2017). Transplanted hPSC-derived ENS progenitors, generated through dual-SMAD inhibition, integrate and migrate extensively within a mouse model of HSCR leading to increased survival (Fattahi et al., 2016). Our work here extends and complements these studies providing further evidence to support the use of hPSCs as a promising platform for the development of cell therapies to treat ENS dysfunction.

## Experimental Procedures

### hPSC Culture and Differentiation

The hESC lines H7 (WA07), H9 (WA09) (Thomson et al., 1998), H9*SOX10*:GFP (Chambers et al., 2012), clinical grade hESC line MasterShef7 and iPSC line SFCi55-ZsGr (Lopez-Yrigoyen et al., 2018) were maintained and NC differentiation performed as described previously (Frith et al., 2018). Enteric neurons were generated by plating day 10 spheres onto Geltrex-coated plates in BrainPhys (STEMCELL Technologies), supplemented with 1× N2, 1× B27, 100 μM ascorbic acid, 10 ng/mL GDNF, and 10 μM DAPT.Use of these Human ES cell lines for this project was approved by the UK Stem Cell Steering Committee, reference SCSC15-14. For full details, see Supplemental Information.

### RNA Extraction, cDNA Synthesis, and qPCR

Detailed methods and primer sequences can be found in the Supplemental Information.

### Immunofluorescence, Image Analysis, and Flow Cytometry

Detailed methods and materials can be found in the Supplemental Information.

### Animals

Animals were maintained, and experiments performed, in accordance with the UK Animals (Scientific Procedures) Act 1986 under license from the Home Office (P0336FFB0) and approved by the University College London Biological Services Ethical Review Process. Animal husbandry at UCL Biological Services was in accordance with the UK Home Office Certificate of Designation.

### *In vivo* Cell Transplantation

Day 6 sorted ZsGreen+/p75++ sorted vagal NC cells were plated into non-adherent plates in N2B27 medium supplemented with 3 μM CHIR99021, 20 ng/mL FGF2, and 10 μM Y27632-dihydrochloride. On day 7, cells were transplanted to the cecum of 4- to 8-week-old immunodeficient Rag2^−/−^;γc^−/−^;C5^−/−^ mice via laparotomy under isofluorane anesthetic. Detailed methods are in the Supplemental Information.

### Whole Mount Gut Immunohistochemistry

Whole mount immunohistochemistry was performed on transplanted cecal and proximal colon segments after cervical dislocation and excision as per McCann et al. (2017). Detailed methods can be found in the Supplemental Information.

## Author Contributions

T.J.R.F., P.W.A., J.O.S.H., C.J.McC., A.J.B., and N.T. conceived the project. T.J.R.F. and C.J.McC. designed, performed, and analyzed the experiments with help from A.G. Z.A.H. and H.D.M. derived the MasterShef7 hESC line. P.W.A., A.J.B., N.T., I.B., A.T., and C.J.McC. provided financial support. T.J.R.F., A.T., C.J.McC., and P.W.A. wrote the manuscript.

## Supplemental Information

Document S1. Supplemental Experimental Procedures, Figures S1–S3, and Tables S1–S3

Document S2. Article plus Supplemental Information

## Footnotes

Supplemental Information can be found online at https://doi.org/10.1016/j.stemcr.2020.07.024.
