# Supplementary material for: Retinoic Acid Accelerates the Specification of Enteric Neural Progenitors from In-Vitro-Derived Neural Crest
Source: Stem Cell Reports. 2020 Aug 27;15(3):557–65. doi: 10.1016/j.stemcr.2020.07.024 (PMC7486303; doi:10.1016/j.stemcr.2020.07.024)
Supplement: Document S1. Supplemental Experimental Procedures, Figures S1–S3, and Tables S1–S3 [file mmc1.pdf]

Stem Cell Reports, Volume *15*

Supplemental Information

Retinoic Acid Accelerates the Specification of Enteric Neural Progenitors from *In-Vitro*-Derived Neural Crest

Thomas J.R. Frith, Antigoni Gogolou, James O.S. Hackland, Zoe A. Hewitt, Harry D. Moore, Ivana Barbaric, Nikhil Thapar, Alan J. Burns, Peter W. Andrews, Anestis Tsakiridis, and Conor J. McCann

FIGURE S1

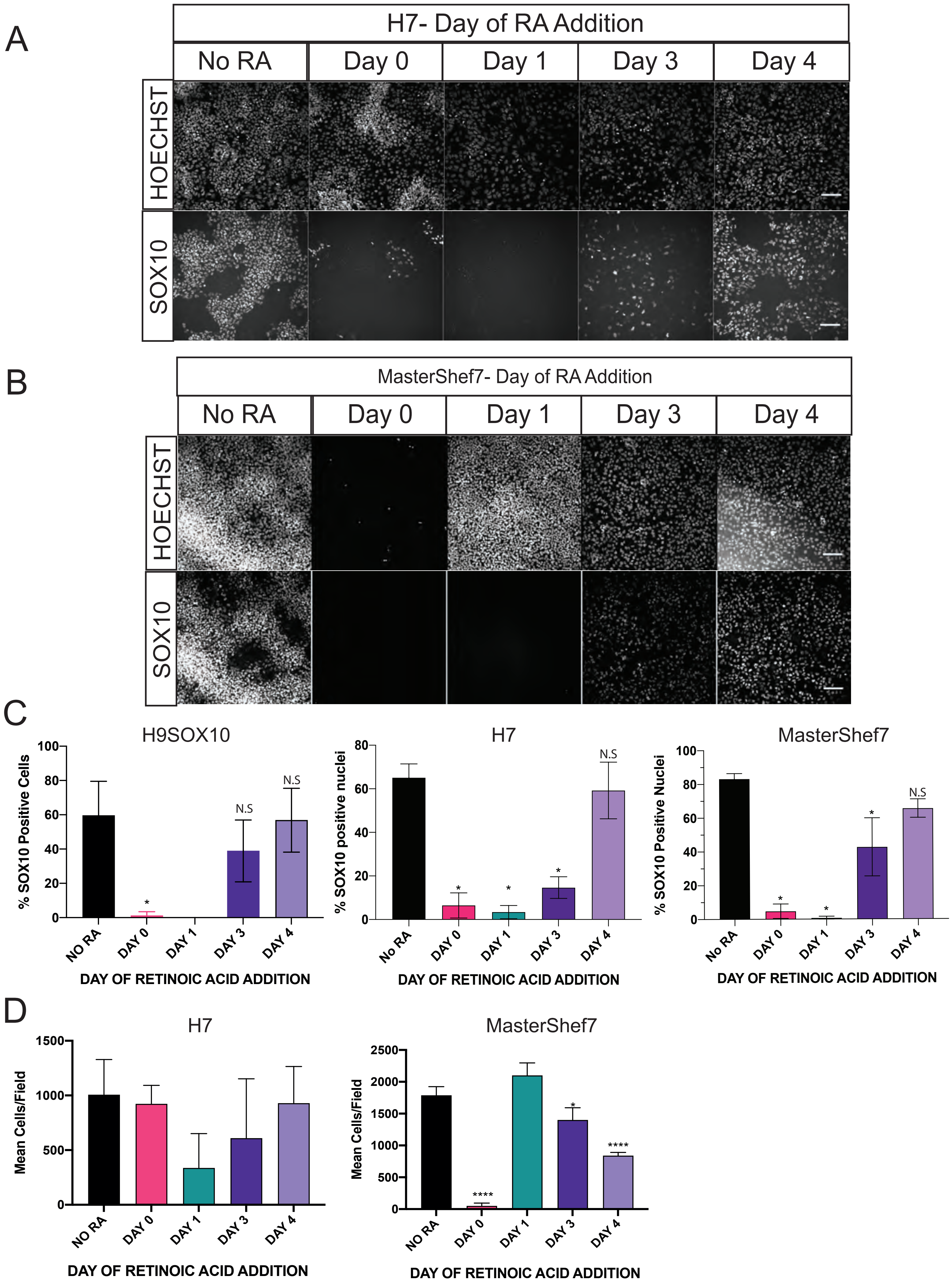
A
H7- Day of RA Addition
No RA
Day 0
Day 1
Day 3
Day 4
HOECHST
SOX10
B
MasterShef7- Day of RA Addition
No RA
Day 0
Day 1
Day 3
Day 4
HOECHST
SOX10
C
H9SOX10
% SOX10 Positive Cells
N.S
N.S
NO RA
DAY 0
DAY 1
DAY 3
DAY 4
DAY OF RETINOIC ACID ADDITION
H7
% SOX10 positive nuclei
N.S
No RA
DAY 0
DAY 1
DAY 3
DAY 4
DAY OF RETINOIC ACID ADDITION
MasterShef7
% SOX10 Positive Nuclei
N.S
No RA
DAY 0
DAY 1
DAY 3
DAY 4
DAY OF RETINOIC ACID ADDITION
D
H7
Mean Cells/Field
NO RA
DAY 0
DAY 1
DAY 3
DAY 4
DAY OF RETINOIC ACID ADDITION
MasterShef7
Mean Cells/Field
****
****
NO RA
DAY 0
DAY 1
DAY 3
DAY 4
DAY OF RETINOIC ACID ADDITION

FIGURE S2

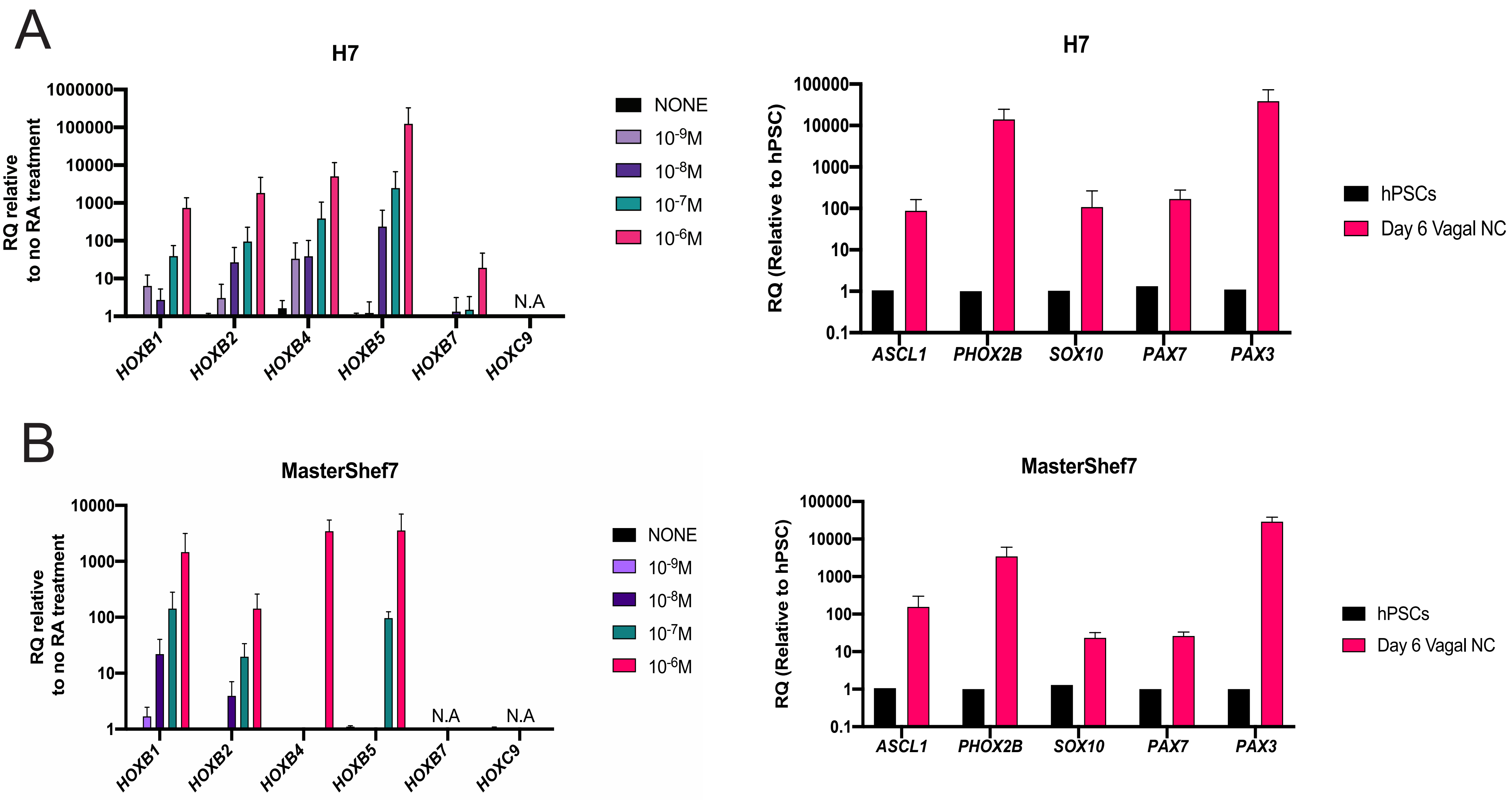
A
H7
RQ relative to no RA treatment
1000000
100000
10000
1000
100
10
1
HOXB1
HOXB2
HOXB4
HOXB5
HOXB7
HOXC9
N.A
NONE
10-9M
10-8M
10-7M
10-6M
H7
RQ (Relative to hPSC)
100000
10000
1000
100
10
1
0.1
ASCL1
PHOX2B
SOX10
PAX7
PAX3
hPSCs
Day 6 Vagal NC
B
MasterShef7
RQ relative to no RA treatment
10000
1000
100
10
1
HOXB1
HOXB2
HOXB4
HOXB5
HOXB7
HOXC9
N.A
N.A
NONE
10-9M
10-8M
10-7M
10-6M
MasterShef7
RQ (Relative to hPSC)
100000
10000
1000
100
10
1
0.1
ASCL1
PHOX2B
SOX10
PAX7
PAX3
hPSCs
Day 6 Vagal NC

FIGURE S3

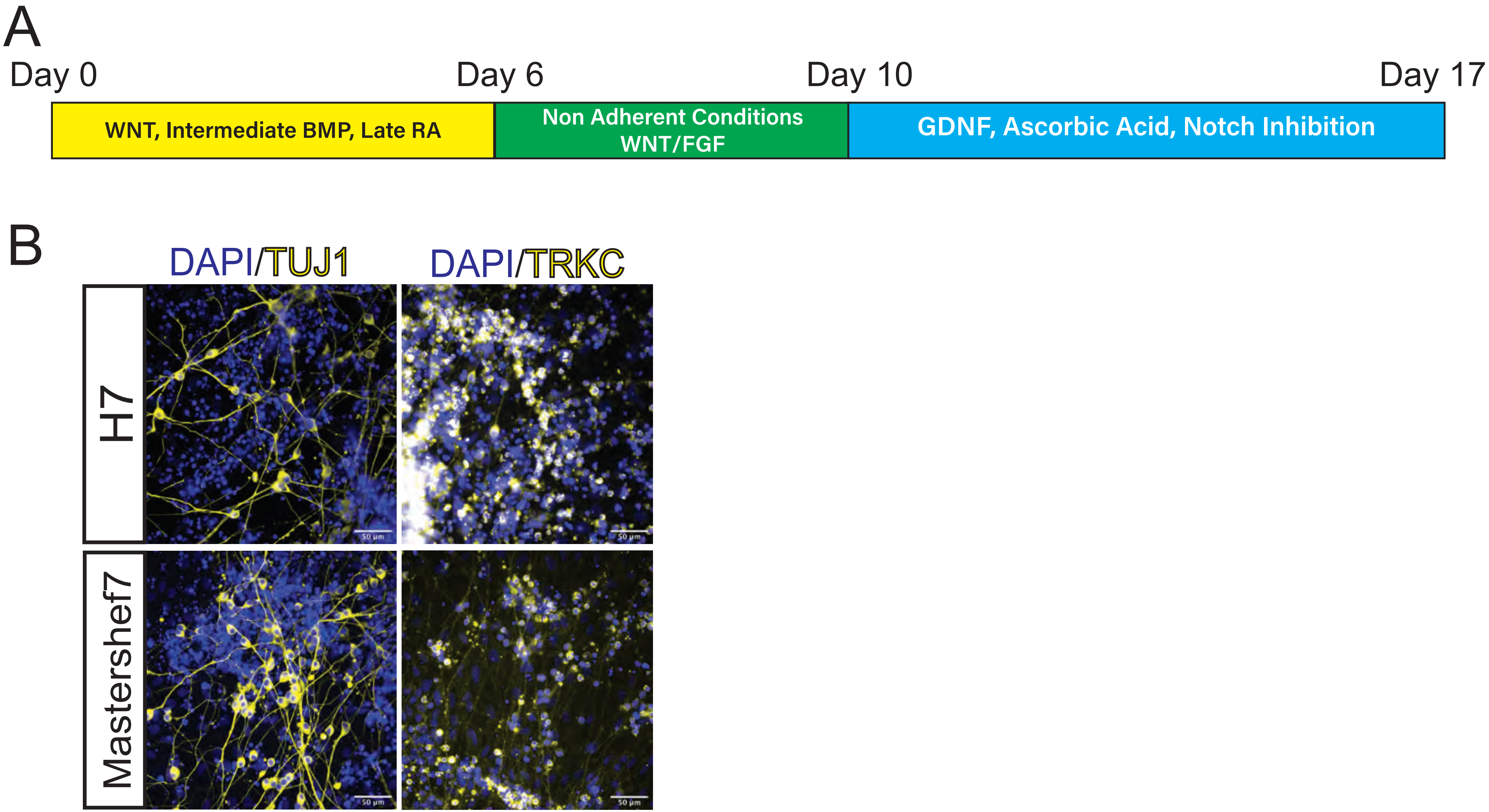
A
Day 0
Day 6
Day 10
Day 17
WNT, Intermediate BMP, Late RA
Non Adherent Conditions
WNT/FGF
GDNF, Ascorbic Acid, Notch Inhibition
B
DAPI/TUJ1
DAPI/TRKC
H7
Mastershef7
50 μm

Supplementary Figure legends

Supplementary Figure 1: RA timing conserved across other hPSC lines.

(A-B) Immunofluorescence images showing SOX10 expression at day 5 after addition of RA at different times during NC differentiation of H7 **(A)** and MasterShef7 **(B)** hPSCs. Scale Bar = 100µm

(C) Quantification of *SOX10*:GFP positive (4 biological repeats) and SOX10 positive cells in H7 (N=3 biological repeats) and MasterShef7 (N=3 biological repeats) after addition of RA at different time points. Bars=mean; error=s.d. *P<0.05, N.S= not significant. One-way ANOVA of cells treated with RA compared to day 5 cells not treated with RA.

(D) Count of the mean number of positive HOECHST nuclei per field for H7 and MasterShef7 following the addition of RA at different timepoints during neural crest differentiation. N=3 independent differentiations. Bars=mean; error=s.d; One-way ANOVA * P<0.05, **** P<0.0001

Supplementary Figure 2: HOX gene induction and early enteric neural marker induction is dependent on the concentration of RA

qPCR analysis showing *HOX* gene and early ENS progenitor marker induction after 6 days of differentiation following RA exposure of **(A)** H7 and **(B)** MasterShef7 hPSCs. N.A = no amplification. N= 3 independent differentiations per line

Supplementary Figure 3: Generation of enteric neurons in 2 further independent hPSC lines.

(A) Enteric neuron differentiation protocol.

(B) Immunofluorescence showing the cells that are positive for TUJ1 and TRKC at day 17 of differentiation. Scale = 50µm. N=3 independent differentiations.

Supplemental Methods

hPSC Culture

The hESC lines H7 (WA07), H9 (WA09) (Thomson et al., 1998), H9:SOX10 (Chambers et al., 2012), clinical grade hESC line MasterShef7 and iPSC line SFCi55-ZsGr (Lopez-Yrigoyen et al., 2018) were grown in mTESR (Stem Cell Technologies # 85850) on 1:100 dilution of Geltrex (ThermoFisher A1413202) in DMEM/F12 (Sigma D6421). Cells were passaged at 80-90% confluency using ReLeSR (Stem Cell Technologies Catalog # 05873). Cells were incubated at 37°C in 5%CO_2. Use of these Human ES cell lines for this project was approved by the UK Stem Cell Steering Committee, reference SCSC15-14.

The clinical grade hESC line, MasterShef7 (https://hpscreg.eu/cell-line/UOSe012-A) was derived at the University of Sheffield, Centre for Stem Cell Biology, under GMP-like conditions in a cleanroom setting (HFEA licence R115-8-A (Centre 0191) and HTA licence 22510).

The frozen embryo was surplus for IVF treatment, donated with fully informed consent, with no financial benefit to the donors and cultured to the blastocyst stage using IVF media (Medicult). Following removal of the trophectoderm using a

dissection laser the embryo was explanted whole onto mitotically inactivated human neonatal fibroblasts (human feeders) in standard KSR/KODMEM (Life Technologies) medium.

MasterShef7 was initially maintained at 37°C under 5% O2 /5% CO2, until established, after which maintenance switched 5% CO2 in air at 37°C. Cultures were passaged using a manual technique, cutting selected colonies under a dissection microscope at an average split ratio of 1:2 every 7 days.

MasterShef7 has been deposited at the UK Stem Cell bank (https://www.nibsc.org/ukstemcellbank) in line with the requirements of the HFEA licence.

Directed Differentiation

For vagal neural crest differentiation, we use a previously described protocol (Frith et al., 2018). hPSCs at approximately 80% confluency were detached using Accutase (Sigma-Aldrich A6964) for 10 minutes at 37°C to generate single cells. Cells were counted manually and plated at 50,000 cells/cm^2 on Geltrex coated plates (ThermoFisher A1413202). Neural Crest differentiation media is comprised of DMEM/F12 (Sigma-Aldrich), supplemented with 1x N2 (ThermoFisher 17502048), NEAA (ThermoFisher 11140050), Glutamax (ThermoFisher 35050061), 1µM CHIR99021 (Tocris 4423), 2µM SB431542 (Tocris 1614/1), 1µM DMH-1 (Tocris 4126/10), 20ng/ml BMP4 (ThermoFisher PHC9533). All-Trans Retinoic Acid (Sigma R2625) was diluted in DMSO. 10µM Y-27632 dihydrochloride (Tocris 1254/1) was added at day 0 until day 2 to assist attachment. For all vagal neural crest induction all-trans Retinoic acid was added at a final concentration of 1µM on day 4 unless specified in the results. Media was changed every other day until day 5/6.

Sphere Formation

Spheres were generated as previously described (Fattahi et al., 2016). Day 6 cells were treated with accutase to form a single cell suspension and replated in a media containing a 1:1 mix of DMEM/F12 (Sigma) with Neurobasal (ThermoFisher 21103049) supplemented with 1x N2, 1x B27, 1x NEAA, 1x Glutamax, 3µM CHIR99021, 10ng/ml FGF2 (R&D systems 233-FB/CF). Sphere media supplemented 10µM of Y-27632 dihydrochloride (Tocris) to ensure sphere formation and left until day 10. One well of a 6 well plate was plated into one well of an Ultra-Low Attachment 6 well plate (Corning 3471) or 6 well plates with a coating of 1% w/v agarose.

Enteric Neuron Differentiation

For enteric neuronal differentiation, day 10 spheres were plated onto Geltrex coated plates in BrainPhys (Stem Cell Technologies 05790) supplemented with 1x N2, 1x B27 (ThermoFisher 17504044), 100µM Ascorbic Acid (Sigma A8960), 10ng/ml GDNF (Peprotech 450-10) and 10µM DAPT (Sigma D5942). Media was changed every other day and once a week supplemented with Vitronectin (ThermoFisher A14700)

Flow Cytometry

A single cell suspension was generated using Accutase as described above. Cells were pelleted and resuspended in FACS buffer (DMEM/10% v/v FCS) at 1x10^6 cells/ml. Gating for positive cells was based on a negative control consisting of cells not carrying a reporter or cells stained with P3X, an antibody from the parent myeloma (KOHLER and MILSTEIN, 1975; Hackland et al., 2017).

RNA extraction, cDNA synthesis & qPCR

RNA was extracted using a Total RNA purification plus kit (Norgen BioTek #48300) per manufacturer's instructions. RNA concentration was measured using a nanodrop (ThermoFisher). RNA was stored at -80° C. cDNA was synthesised using the High-Capacity cDNA Reverse Transcription kit (ThermoFisher 4368813) and stored at -20°C.

qPCR was performed on QuantStudio 12K Flex thermocycler (Applied Biosystems). CT values were calculated against GAPDH for each sample. Relative quantities calculated using the -2^^CT method. Primer sequences can be found in Tables 2 & 3.

Immunofluorescence & Image Analysis

Cells were fixed with 4% PFA for 10 minutes at room temperature and washed 3 times with 1x PBS (no Mg^{2+}/ Ca^{2+}). Cells were permeabilised and blocked with 1x PBS (no Mg^{2+}/ Ca^{2+}) supplemented with 10% FCS, 0.1% BSA and 0.3% Triton-X 100 for 1 hour at room temperature. Primary antibodies were diluted in permeabilisation buffer and incubated at 4°C overnight. Secondary antibodies were diluted in permeabilization buffer and stained in the dark at 4°C for 1 hour. Nuclei were counterstained with Hoechst 33342 (ThermoFisher H3570). Images were taken on an InCell Analyser 2500 (GE Healthcare) and quantified using custom made pipelines on CellProfiler 2.2 (Carpenter et al., 2006) as per (Frith et al., 2018).

Table 1: Antibodies used in the study

	Antibody	Species	Source	Dilution
In Vitro	SOX10	Rabbit	Cell Signalling Technology (D5V9L) #89356	1:500
	RET	Rabbit	Abcam ab134100	1:1000
	TUJ1	Mouse	Abcam ab78078	1:1000
	TRKC	Rabbit	Cell Signalling Technology (C44H5) #3376	1:1000
	PERIPHERIN	Rabbit	Millipore AB1530	1:100
	P3X	Mouse	In house myeloma P3X63Ag8 (Kohler & Milstein 1975)	1:10
	p75	Mouse	In house hybridoma Clone ME20.4 (Ross et al., 1984)	1:20
	CD49d	Mouse	BioLegend 304302 Clone 9F10	1:100
Transplants	TUJ1	Mouse	BioLegend MMS-435P	1:500
	GFAP	Rabbit	Millipore AB5804	1:500
	nNOS	Rabbit	Invitrogen 61-7000	1:400
	vAChT	Goat	ThermoFisher Scientific OSH00003W	1:200
	DAPI		Sigma D8417	1:1000

Table 2: Primers and UPL probes used in study

Gene	Forward	Reverse	Roche UPL Probe
GAPDH	agccacatcgctcagacac	gcccaatacgaccaaatcc	60
HOXB1	ccagctagggggcttgtc	atgctgcggaggatatgg	39
HOXB2	aatccgccacgtctcctt	gctgcgtgttggtgtaagc	70
HOXB4	ctggatgcgcaaagttcac	agcggttgtagtgaaattcctt	62
HOXB5	aagcttcacatcagccatga	cggttgaagtggaactccttt	1
HOXB7	ctacccctggatgcgaag	caggtagcgattgtagtgaaattct	1
HOXC9	gcagcaagcacaaagagga	cgtctggtacttggtgtaggg	85
SOX10	ggctcccccatgtcagat	ctgtcttcggggtggttg	21
PAX3	aggaggccgacttggaga	cttcatctgattggggtgct	13
PAX7	gaaaacccaggcatgttcag	gcggctaatcgaactcactaa	66
ASCL1	cgacttcaccaactggttctg	atgcaggttgtgcgatca	38
PHOX2A	cactaccccgacatttacacg	gctcctgtttgcggaactt	17
PHOX2B	ctaccccgacatctacactcg	ctcctgcttgcgaaacttg	17
SST	accccagactccgtcagttt	acagcagctctgccaagaag	38
CHAT	cagccctgatgccttcat	cagtcttcgatggagcctgt	78
TH	acgccaaggacaagctca	agcgtgtacgggtcgaact	42
HTR2a	tgatgtcacttgccatagctg	caggtaaatccagactgcacaa	3
GFRA1	caccattgccctgaaagaat	cgcttttaggggttcaggtc	36

Table 3: Taqman Gene Expression Assays for Figure 3G. Thermo Fisher Scientific Cat # 4331182

Gene	Assay ID
GAPDH	Hs03929097_g1
PLP1	Hs00166914_m1
S100β	Hs00902901_m1
FABP7	Hs00361424_g1
ERBB3	Hs00176538_m1
SOX10	Hs00366918_m1

In vivo cell transplantation

Day 6 P75++/ZsGreen+ cells were purified and grown as spheres for one day as outlined above and transplanted the following day (day 7 of differentiation). Briefly, the caecum was exposed and ZsGreen+ spheres, containing 1 million cells each, were subsequently transplanted to the serosal aspect of the caecum by mouth pipette, using a pulled glass micropipette. Each transplanted tissue typically received 3 ZsGreen+ spheres which were manipulated on the surface of the caecum with the bevel of a 30G needle to ensure correct positioning. Transplanted Rag2$^{-/-}$;γc$^{-/-}$;C5$^{-/-}$mice were typically maintained for either 2 weeks (N=2), 4 weeks (N=9) or 3 months (N=4) post-transplantation, before sacrifice and removal of the caecum and proximal colon for analysis.

Animals

Rag2$^{-/-}$;γc$^{-/-}$;C5$^{-/-}$ mice, which lack innate immunity, and are deficient in all lymphocytes (Cooper et al., 2003; Silva-Barbosa et al., 2005), were used as recipients for all transplants.

Wholemount Gut Immunohistochemistry

Tissues were fixed in ice cold 4% PFA for 45 min at 22°C. After fixation, tissues were washed for 24h in 1x PBS at 4°C. Cells were permeabilised and blocked with 1x PBS supplemented with 1% Triton X-100 and 10% sheep serum. Primary antibodies were diluted in permeabilisation buffer and incubated at 4°C for 48h. Secondary antibodies were diluted in permeabilisation buffer and stained in the dark for one hour at 22°C. Nuclei were counterstained with DAPI (Sigma). Before mounting, tissues were washed thoroughly in 1x PBS for 2h at 22 °C. Tissues were examined using a LSM710 Meta confocal microscope (Zeiss). Confocal micrographs of whole mounts were digital composites of the Z-series of scans (0.5-1μm optical sections, 10–50μm thick).

Supplemental References

Carpenter, A.E., Jones, T.R., Lamprecht, M.R., Clarke, C., Kang, I.H., Friman, O., Guertin, D.A., Chang, J.H., Lindquist, R.A., Moffat, J., Golland, P. and Sabatini, D.M. 2006. CellProfiler: image analysis software for identifying and quantifying cell phenotypes. *Genome biology*. **7**. R100.

Chambers, S.M., Qi, Y., Mica, Y., Lee, G., Zhang, X.-J., Niu, L., Bilsland, J., Cao, L., Stevens, E., Whiting, P., Shi, S.-H. and Studer, L. 2012. Combined small-molecule inhibition accelerates developmental timing and converts human pluripotent stem cells into nociceptors. *Nat Biotechnol*. **30**. 715–720.

Cooper, R.N., Thiesson, D., Furling, D., Di Santo, J.P., Butler-Browne, G.S. and Mouly, V. 2003. Extended amplification in vitro and replicative senescence: key factors implicated in the success of human myoblast transplantation. *Human gene therapy*. **14**. 1169–1179.

Fattahi, F., Steinbeck, J.A., Kriks, S., Tchieu, J., Zimmer, B., Kishinevsky, S., Zeltner, N., Mica, Y., El-Nachef, W., Zhao, H., de Stanchina, E., Gershon, M.D., Grikscheit, T.C., Chen, S. and Studer, L. 2016. Deriving human ENS lineages for cell therapy and drug discovery in Hirschsprung disease. *Nature*. **531**.105–109.

Frith, T.J., Granata, I., Wind, M., Stout, E., Thompson, O., Neumann, K., Stavish, D., Heath, P.R., Ortmann, D., Hackland, J.O., Anastassiadis, K., Gouti, M., Briscoe, J., Wilson, V., Johnson, S.L., Placzek, M., Guarracino, M.R., Andrews, P.W. and Tsakiridis, A. 2018. Human axial progenitors generate trunk neural crest cells in vitro. *Elife*. **7**. 134.

Hackland, J.O.S., Frith, T.J.R., Thompson, O., Marin Navarro, A., García-Castro, M.I., Unger, C. and Andrews, P.W. 2017. Top-Down Inhibition of BMP Signaling Enables Robust Induction of hPSCs Into Neural Crest in Fully Defined, Xeno-free Conditions. *Stem Cell Reports*. **9**. 1043–1052.

KOHLER, G. and MILSTEIN, C. 1975. Continuous cultures of fused cells secreting antibody of predefined specificity. *Nature*. **256**. 495–497.

Lopez-Yrigoyen, M., Fidanza, A., Cassetta, L., Axton, R.A., Taylor, A.H., Meseguer-Ripolles, J., Tsakiridis, A., Wilson, V., Hay, D.C., Pollard, J.W. and Forrester, L.M. 2018. A human iPSC line capable of differentiating into functional macrophages expressing ZsGreen: a tool for the study and in vivo tracking of therapeutic cells. *Philosophical transactions of the Royal Society of London. Series B, Biological sciences*. **373**(1750).

Ross, A.H., Grob, P., Bothwell, M., Elder, D.E., Ernst, C.S., Marano, N., Ghrist, B.F., Slemp, C.C., Herlyn, M. and Atkinson, B. 1984. Characterization of nerve growth factor receptor in neural crest tumors using monoclonal antibodies. *Proc Natl Acad Sci U S A*. **81**. 6681–6685.

Silva-Barbosa, S.D., Butler-Browne, G.S., Di Santo, J.P. and Mouly, V. 2005. Comparative analysis of genetically engineered immunodeficient mouse strains as recipients for human myoblast transplantation. *Cell transplantation*. **14**. 457–467.

Thomson, J.A, Itskovitz-Eldor, J., Shapiro, S.S, Waknitz, M. A., Swiergiel, J.J., Marshall, V. S., Jones, J. M., 1998. Embryonic Stem Cell Lines Derived from Human Blastocysts. *Science*. **282**. 1145–1147.
