# Supplementary material for: Retinoic Acid Accelerates the Specification of Enteric Neural Progenitors from In-Vitro-Derived Neural Crest
Source: Stem Cell Reports. 2020 Aug 27;15(3):557–65. doi: 10.1016/j.stemcr.2020.07.024 (PMC7486303; doi:10.1016/j.stemcr.2020.07.024)
Supplement: Document S2. Article plus Supplemental Information [file mmc2.pdf]

Stem Cell Reports
Report
OPEN ACCESS

# Retinoic Acid Accelerates the Specification of Enteric Neural Progenitors from *In-Vitro*-Derived Neural Crest

Thomas J.R. Frith,[1,8,*] Antigoni Gogolou,[1] James O.S. Hackland,[2] Zoe A. Hewitt,[1] Harry D. Moore,[1] Ivana Barbaric,[1] Nikhil Thapar,[3,4,5,6] Alan J. Burns,[3,7] Peter W. Andrews,[1] Anestis Tsakiridis,[1,*] and Conor J. McCann[3,*]
[1]University of Sheffield, Department of Biomedical Science, Sheffield, UK
[2]The Center for Stem Cell Biology, Memorial Sloan Kettering Cancer Center, New York, USA
[3]Stem Cells and Regenerative Medicine, UCL Great Ormond Street Institute of Child Health, London, UK
[4]Neurogastroenterology and Motility Unit, Great Ormond Street Hospital, London, UK
[5]Department of Gastroenterology, Hepatology and Liver Transplant, Queensland Children's Hospital, Brisbane, Australia
[6]Prince Abdullah Ben Khalid Celiac Research Chair, College of Medicine, King Saud University, Riyadh, KSA
[7]Department of Clinical Genetics, Erasmus University Medical Center, Rotterdam, The Netherlands
[8]Present address: Francis Crick Institute, 1 Midland Rd, London, UK
*Correspondence: tom.frith@crick.ac.uk (T.J.R.F.), a.tsakiridis@sheffield.ac.uk (A.T.), conor.mccann@ucl.ac.uk (C.J.M.)
https://doi.org/10.1016/j.stemcr.2020.07.024

## SUMMARY

The enteric nervous system (ENS) is derived primarily from the vagal neural crest, a migratory multipotent cell population emerging from the dorsal neural tube between somites 1 and 7. Defects in the development and function of the ENS cause a range of enteric neuropathies, including Hirschsprung disease. Little is known about the signals that specify early ENS progenitors, limiting progress in the generation of enteric neurons from human pluripotent stem cells (hPSCs) to provide tools for disease modeling and regenerative medicine for enteric neuropathies. We describe the efficient and accelerated generation of ENS progenitors from hPSCs, revealing that retinoic acid is critical for the acquisition of vagal axial identity and early ENS progenitor specification. These ENS progenitors generate enteric neurons *in vitro* and, following *in vivo* transplantation, achieved long-term colonization of the ENS in adult mice. Thus, hPSC-derived ENS progenitors may provide the basis for cell therapy for defects in the ENS.

## INTRODUCTION

The enteric nervous system (ENS) is the largest branch of the peripheral nervous system and consists of an extensive network of neurons and glia controlling critical intestinal functions, such as motility, fluid exchange, gastric acid/hormone secretion, and blood flow (reviewed in Sasselli et al., 2012). In amniotes, the ENS is derived predominantly from the vagal neural crest (NC), a multipotent cell type specified at the neural plate border between somites 1 and 7. The vagal NC contributes to structures in various other organs, such as the heart, thymus, and lungs (Hutchins et al., 2018; Le Douarin et al., 2004; Simkin et al., 2018). After delaminating from the dorsal neural tube, vagal NC cells migrate and enter the foregut where enteric neural progenitors colonize the developing gut in a rostro-caudal direction. Determinants of ENS progenitor migration, proliferation, and differentiation include the RET-GDNF (Durbec et al., 1996) and endothelin-3-EDNRB (Baynash et al., 1994; Hosoda et al., 1994) signaling pathways and the transcription factors *SOX10*, *PHOX2B*, and *ASCL1* (Bondurand et al., 2006; Elworthy et al., 2005; Memic et al., 2016). However, the signals that shape early ENS identity within vagal NC precursors remain less well defined.

Vagal NC cells express members of the HOX paralogous groups (PG) 3–5 (Diman et al., 2011; Fu et al., 2003; Kam and Lui, 2015) and are patterned mainly by the action of somite-derived retinoic acid (RA) signaling, which acts by "posteriorizing" cranial $HOX^-$ NC progenitors (Frith et al., 2018; Ishikawa and Ito, 2009; Stuhlmiller and García-Castro, 2012). *In vivo* studies implicate RA in the specification of downstream vagal NC derivatives (El Robrini et al., 2016; Niederreither et al., 2001, 2003), particularly the ENS where RA signaling components control progenitor migration and proliferation (Niederreither et al., 2003; Uribe et al., 2018).

hPSCs offer an attractive approach for dissecting early cell fate decisions. To date, few studies have described the generation of ENS progenitors and neurons from PSCs indicating that these cell populations can be used to model and treat enteric neuropathies, such as Hirschsprung disease (HSCR) (Fattahi et al., 2016; Lai et al., 2017; Li et al., 2016; Workman et al., 2016). These protocols rely on transforming growth factor β/BMP inhibition followed by exposure to WNT, BMP, and RA to form vagal NC, yielding ENS progenitors after 10–15 days in culture (Fattahi et al., 2016; Workman et al., 2016). However, the precise timing and concentration of RA signaling that control the positional identity of NC cells has not been clearly defined. Moreover, it is not yet clear whether RA imparts an early enteric neural identity in hPSC-derived vagal NC or acts solely as a positional specifier.

We previously described the efficient and robust production of NC cells from hPSCs (Frith et al., 2018; Hackland


This is an open access article under the CC BY license (http://creativecommons.org/licenses/by/4.0/).

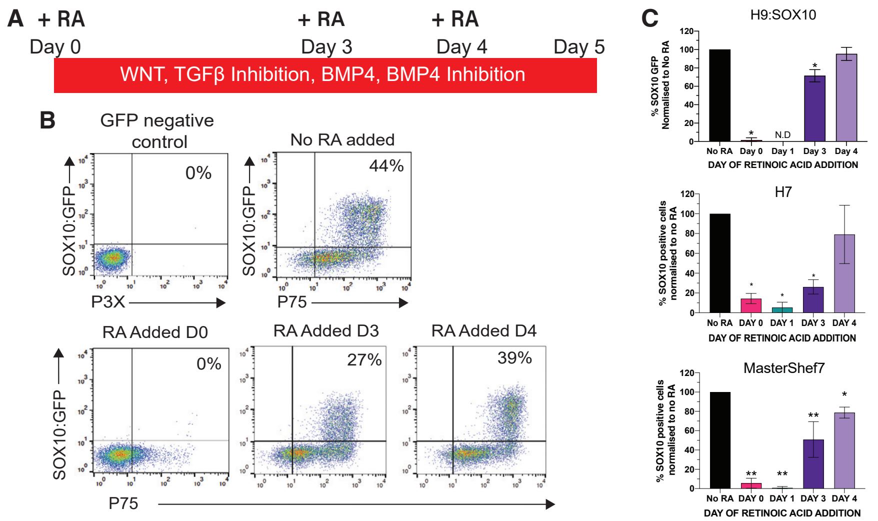


**Figure 1. RA Affects NC Specification in a Time-Dependent Manner**

(A) Schematic of NC differentiation protocol and time points corresponding to addition of all-*trans* RA.

(B) FACS plots showing *SOX10*:GFP and p75 expression at day 5 after RA addition at indicated time points during NC differentiation.

(C) Percentage of cells expressing SOX10 in three hPSC lines following FACS or immunofluorescence. Graphs show percentage of SOX10+ cells normalized to cells not treated with RA. Bars = mean; error = SD. N = 4 independent differentiations for *SOX10*:GFP. N = 3 independent differentiations for H7 and MasterShef7. $^{*}p < 0.05$, $^{**}p < 0.01$; one-way ANOVA.

et al., 2017), which can acquire a vagal axial identity following exposure to RA (Frith et al., 2018). This method overcame variations in NC induction due to variable levels of endogenous BMP, typical of hPSC cultures, by using Top-down inhibition (Hackland et al., 2017) in which a saturating level of exogenous BMP supplements endogenous BMP and the signaling is precisely modulated by a BMP inhibitor. Using this system, we show that RA acts in a dose-dependent manner on pre-specified NC precursors to induce vagal *HOX* genes and direct the accelerated production of ENS progenitors that generate enteric neurons *in vitro* and colonize the ENS of adult mice following long-term transplantation. Our findings provide an efficient platform for *in vitro* modeling of human ENS development and disease, and development of cell therapy-based approaches for the treatment of such conditions.

## RESULTS

### The Timing of RA Signaling Affects NC Specification *In Vitro*

We previously showed that RA treatment of cranial NC precursors induces a vagal axial identity, defined by expression of HOX PG members 1–5 (Frith et al., 2018). To identify the developmental time window during which RA imparts a vagal identity without perturbing NC specification, we supplemented 1 μM all-*trans* RA at different stages of NC differentiation (Figure 1A). The NC markers p75 and *SOX10* were assessed by flow cytometry in a *SOX10*:GFP reporter hPSC line (Chambers et al., 2012). Adding RA at day 0 of differentiation did not yield any *SOX10*:GFP+/p75+ cells at day 5, while addition of RA at days 3 or 4 of differentiation saw similar levels of *SOX10*:GFP+/p75+ cells compared with untreated cells (Figures 1B and 1C). Immunostaining for SOX10 in two other hPSC lines (H7 and MasterShef7) confirmed the same temporal effect of RA on NC differentiation from hPSCs (Figures 1C and S1). While not affecting the efficiency of NC differentiation, RA did cause a variable reduction of the number of cells at day 6 of differentiation (Figure S1D), indicating low levels of RA toxicity. These data suggest that early RA signaling perturbs NC induction from hPSCs, while late addition of RA changes the axial identity of cells committed to NC fate.

### RA Induces Both Vagal and Enteric Neural Progenitor Identities in a Dose-Dependent Manner

RA induces *HOX* gene expression in a dose-dependent manner *in vitro* (Okada et al., 2004; Simeone et al., 1990) and *in vivo* (Papalopulu et al., 1991). To examine how levels of RA signaling shape the axial identity of hPSC-derived NC cells, we treated day 4 HOX$^{-}$ NC precursors with $10^{-9}$ M (1 nM) to $10^{-6}$ M (1 μM) RA and examined the expression of HOX and NC/ENS progenitor genes (Figure 2). *HOXB1* and *B2*, were induced by all concentrations of RA in a dose-dependent manner, while HOX genes marking vagal NC (*HOXB4*, *B5*, and *B7*) were only induced by higher RA concentrations (Figures 2B and S2), consistent with previous findings (Okada et al., 2004). No *HOXC9* expression was observed with any RA concentration, consistent with findings that truncal NC identity is mediated by WNT/FGF signaling (Abu-Bonsrah et al., 2018; Frith et al., 2018; Hackland et al., 2019; Lippmann et al., 2015).

Expression of the NC markers *SOX10*, *PAX7*, and *PAX3* was unaffected by the levels of RA (Figures 2C, 2D, and S2) in line with our previous observations (Figure 1). The highest concentrations of RA elicited higher expression of *ASCL1* and *PHOX2B* (Figure 2D) that mark peripheral

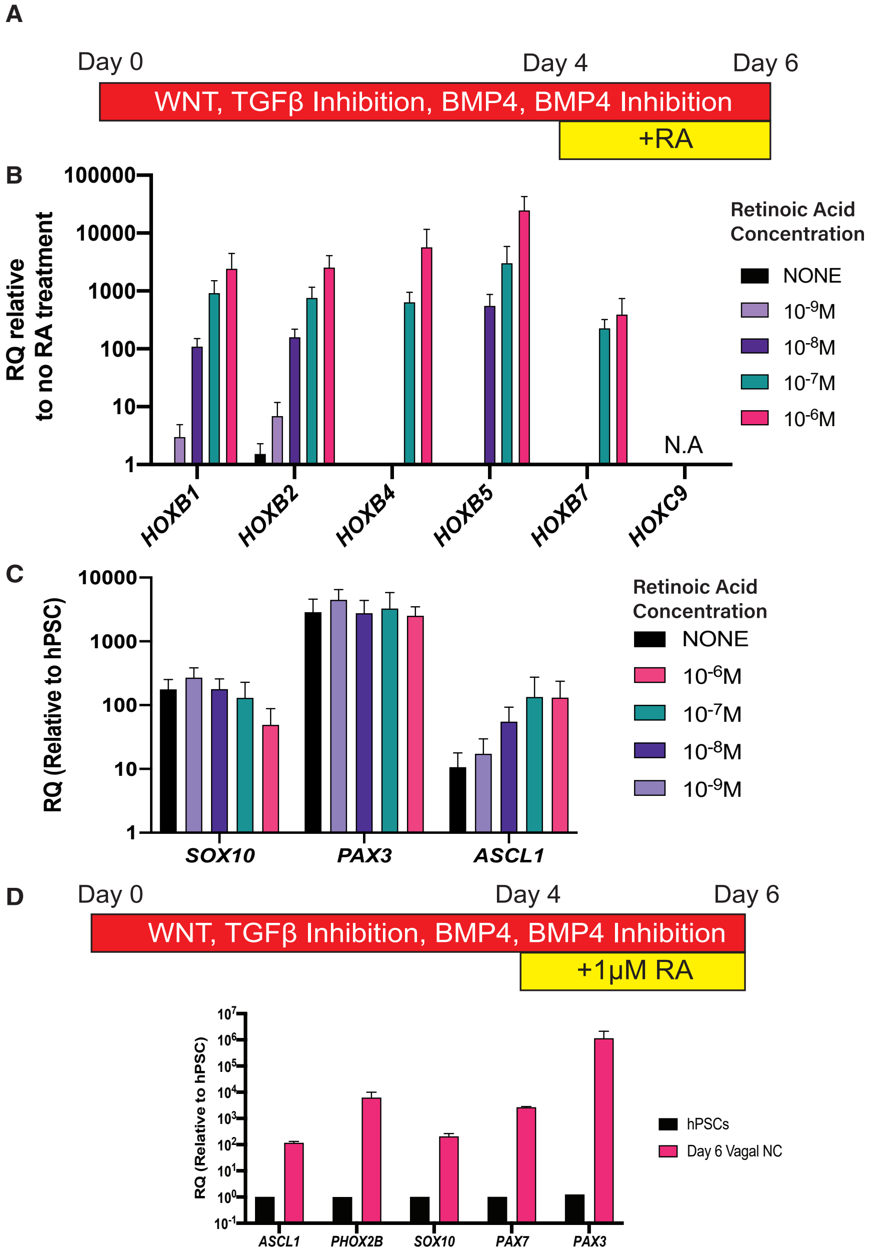


**Figure 2. RA Induces a Vagal and ENS Progenitor Identity In a Dose-Dependent Fashion**

(A) Differentiation protocol to pattern hPSC-derived NC cells.

(B and C) qPCR plots showing the induction of *HOX* genes (B) and NC/ENS markers (C) at day 6 relative to non-RA-treated *HOX negative* cells after exposure to different concentrations of RA. Bar = mean, error bars = SD, N = 3 independent differentiations of *SOX10*:GFP hPSCs. N.A., no amplification.

(D) qPCR plots showing NC/enteric neural precursor markers in day 6 cells after 2 days exposure of 1 μM RA. Bar = mean, error bars = SD, N = 3 independent differentiations.

nervous system precursors, including migrating ENS progenitors (Blaugrund et al., 1996; Elworthy et al., 2005; Lo et al., 1991). These results indicate that acquisition of a vagal axial identity and ENS progenitor specification in NC progenitors are tightly coupled events dependent on RA signaling.

### RA-Induced Vagal NC/ENS Progenitors Generate Putative Enteric Neurons *In Vitro*

To test if day 6 vagal NC cells possess ENS progenitor potential, we tested their ability to form enteric neurons *in vitro*. Day 6 vagal NC cells were cultured in the presence of WNT/FGF in non-adherent conditions (Figure 3A), as described previously (Fattahi et al., 2016). Spheres retained *SOX10*:GFP expression, immunoreactivity of ENS precursor markers p75 and CD49d, and vagal NC and *HOX* gene expression (Figure 3D) after 4 days of culture (Figures 3B and 3C). At day 10, spheres were re-plated in conditions promoting enteric neuron differentiation (Figure 3E) (Fattahi et al., 2016; Okamura and Saga, 2008; Theocharatos et al., 2013). At day 17, we observed cells expressing the enteric neuronal markers TUJ1, RET,

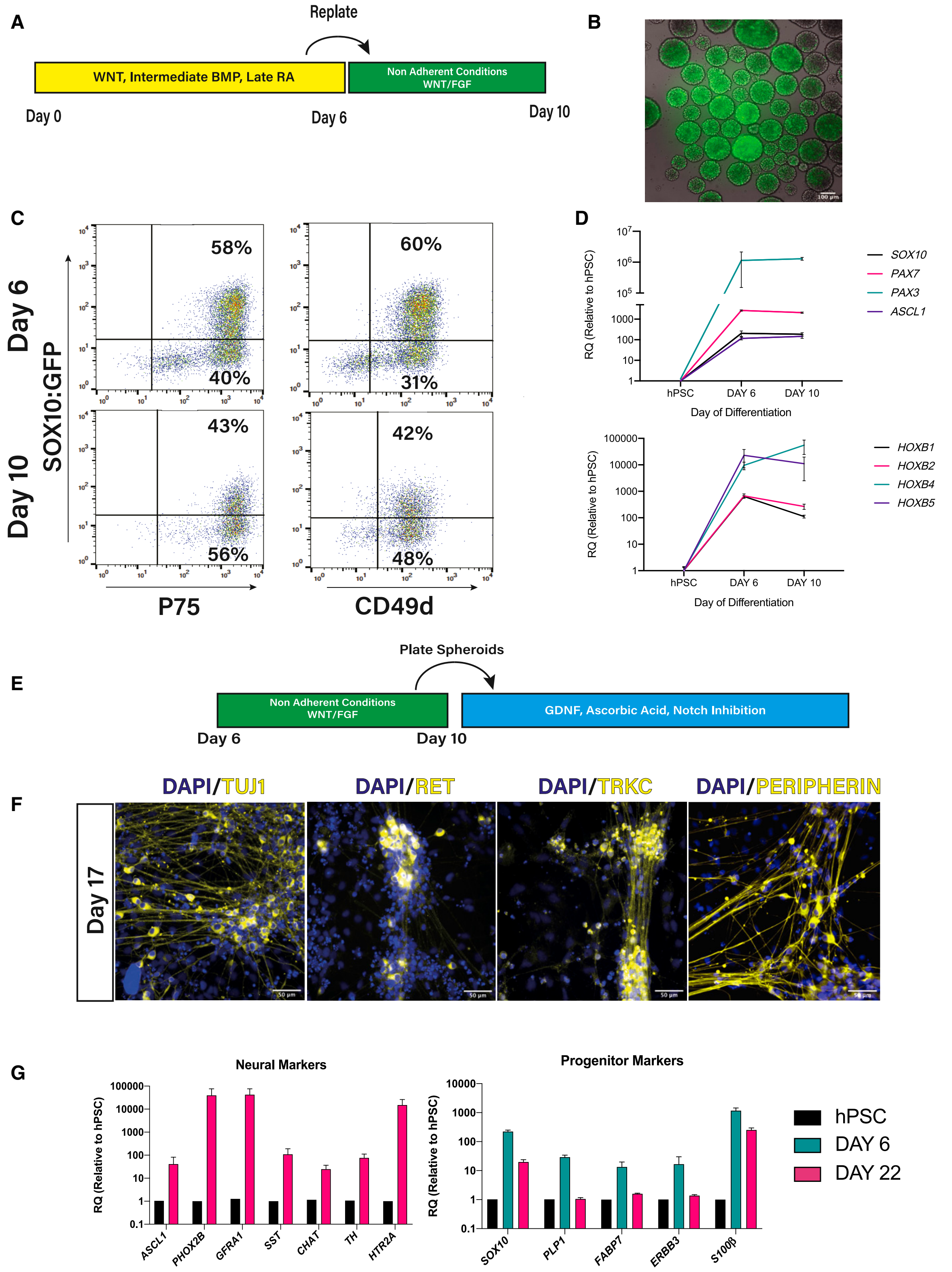


(legend on next page)

TRKC, and PERIPHERIN (Figure 3F). Similar results were obtained with two other hPSC lines (Figure S3). *ChAT*, *HTR2A*, *TH*, and *ASCL1* expression at day 22 further indicated the presence of early enteric neurons. The expression of glial/neuronal progenitor markers *SOX10* and *S100β* (Lasrado et al., 2017) were also detected in day 22 cultures, but were found to be reduced from day 6. *PLP1 ERBB3* and *FABP7* were expressed in day 6 ENS precursors, but switched off by day 22, consistent with the neurogenic effect of NOTCH inhibition (Figure 3G). These observations suggest that RA-induced NC cells can give rise to enteric neurons *in vitro*.

### RA-Induced Vagal NC/ENS Progenitors Colonize the Adult Mouse ENS *In Vivo*

To assess the developmental potential of hPSC-derived vagal NC/ENS progenitors *in vivo*, we performed transplantation to the cecum of adult immunodeficient $Rag2^{-/-};\gamma c^{-/-};C5^{-/-}$ mice. To track transplanted cells, we used the PSC line SFCi55-ZsGr, which contains a constitutive ZsGreen reporter in the *AAVS* locus (Lopez-Yrigoyen et al., 2018). We generated spheres from day 6 ZsGreen+/p75++-sorted putative ENS progenitors. The next day, ZsGreen+/P75++ cells were transplanted to the serosal aspect of the cecum in adult (4–8 weeks) immunodeficient $Rag2^{-/-};\gamma c^{-/-};C5^{-/-}$ mice and analyzed for integration and differentiation at timed intervals (Figures 4A and 4B). Two weeks post-transplantation TUJ1+ ZsGreen+ cells (Figure 4C, left; arrowheads), were observed at the serosal aspect within the cecum and proximal colon (N = 2/2 mice). At 4 weeks post-transplantation ZsGreen+ cells were again observed on the serosal surface and within the tunica muscularis at the level of the myenteric plexus (N = 8/9 mice). Within the tunica muscularis, ZsGreen+ cells co-expressed TUJ1 both within myenteric ganglia-like structures and as intramuscular neurons (Figure 4C, right). We also detected ZsGreen+ cells at the level of the myenteric plexus, which were positive for the glial marker GFAP (Figure 4C, right). After 3 months, ZsGreen+ cells could be found across the gut wall both within individual myenteric ganglia (Figure 4D, left) and the submucosa, surrounding cryptal structures (N = 3/4 mice) (Figure 4D, right). Furthermore, there were ZsGreen+ cells that had differentiated into enteric neuronal subtypes expressing either neuronal nitric oxide synthase (nNOS) (Figure 4D, left) or vesicular acetylcholine transporter (vAChT) (Figure 4D, right). At this time, donor cell coverage averaged 2.8 ± 1.8 $mm^2$ compared with 0.05 ± 0.02 $mm^2$ after 2 weeks post-transplantation (N = 2 mice/time point). These results suggest that hPSC-derived ENS progenitors integrate within recipient gut and are maintained long-term, differentiating to multiple neuronal subtypes and glia.

## DISCUSSION

We describe a differentiation system utilizing RA to drive the concomitant induction of both a vagal and an ENS progenitor identity from hPSCs. Top-down inhibition produces an intermediate level of BMP signaling that, in combination with WNT, robustly and efficiently generates NC after 5–6 days *in vitro* (Frith et al., 2018; Hackland et al., 2017), which go on to express vagal level HOX genes after RA signaling. This is quicker than previously published protocols that yield ENS progenitors after 10–15 days (Fattahi et al., 2016; Workman et al., 2016). We also report the induction of *ASCL1* and *PHOX2B* shortly after the addition of RA to NC precursors, which, combined with *SOX10* and p75 expression, is consistent with ENS progenitor identity (Figure 2). We previously showed that RA treatment of NC precursors also induces markers of cardiac and posterior cranial NC alongside ENS progenitor markers during vagal NC specification (Frith et al., 2018), suggesting that axial identity and cell fate are inter-linked.

Previous studies reveal a role for RA signaling in promoting ENS progenitor migration, proliferation, and differentiation (Niederreither et al., 2003; Simkin et al., 2013; Uribe et al., 2018). RA may control these processes through vagal HOX genes such as *HOXB3*, *HOXB5*, and TALE family cofactors, which regulate ENS development (Chan et al., 2005; Kam and Lui, 2015; Uribe and Bronner, 2015; Uribe et al., 2018) by inducing *Ret* (Zhu et al., 2014) and preventing apoptosis (Kam et al., 2014).

Our differentiation strategy rapidly and robustly yields a well-defined progenitor cell population that can generate

**Figure 3. Day 6 Enteric Neural Precursors Can Generate Putative Enteric Neurons *In Vitro***

(A) Schematic of non-adherent culture conditions.

(B) Day 8 NC spheres containing *SOX10*:GFP+ cells.

(C) FACS plots of *SOX10*:GFP and p75/CD49d expression in non-adherent conditions from day 6 to 10.

(D) qPCR showing vagal NC/early ENS markers at days 6 and 10 of differentiation. Bars = mean; error = SD. N = 3 independent differentiations.

(E) Enteric neuron differentiation conditions.

(F) Immunofluorescence for enteric neuron markers at day 17 of differentiation. Scale bar = 50 μm.

(G) qPCR analysis of enteric neuron and progenitor markers at day 22 of differentiation. Bars = mean; error = SD. N = 3 independent differentiations in SOX10:GFP hPSCs.

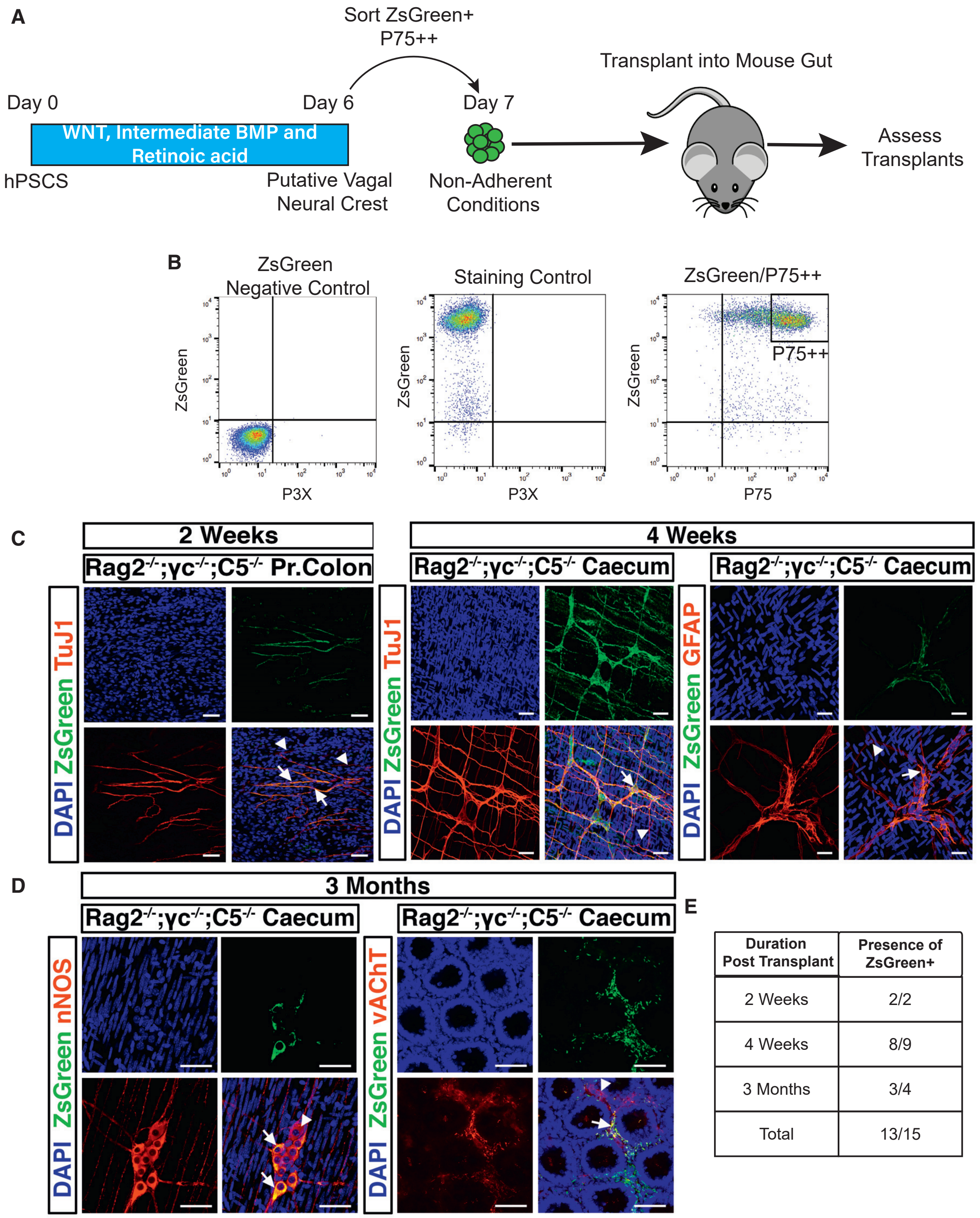


| Duration Post Transplant | Presence of ZsGreen+ |
|---|---|
| 2 Weeks | 2/2 |
| 4 Weeks | 8/9 |
| 3 Months | 3/4 |
| Total | 13/15 |

**Figure 4. hPSC-Derived Enteric Neuronal Precursors Integrate into the Mouse ENS after Transplantation**
(A) Schematic of procedures for transplantation of hPSC-derived ENS progenitors.
(B) Sorting strategy to isolate *in-vitro*-derived ZsGreen+/p75++-labeled putative ENS progenitors.
(C) Whole-mount images of gut tissue corresponding to the indicated regions obtained at 2 and 4 weeks post-transplantation. Arrows show ZsGreen+ cells that are positive for TUJ1 among endogenous TUJ1+ neurons (arrowheads), and glial marker GFAP after immunostaining. Pr. Colon, proximal colon. Scale bar = 50 μm.

*(legend continued on next page)*

enteric neurons *in vitro* (Figure 3). To test their potential as a cellular donor to treat enteric neuropathies, we transplanted our ENS progenitors into the gut of immunodeficient Rag2$^{-/-}$;γc$^{-/-}$;C5$^{-/-}$ mice. This eliminated the requirement for chemical immunosuppression, allowing long-term study of donor cell survival and integration within a "normal" host ENS microenvironment. Crucially, we found that the hPSC-derived neurons were present within endogenous ENS ganglia of adult mice up to 3 months post-transplantation (N = 3/4), expressing the same markers (nNOS and vAChT) as host neuronal populations (Figure 4D). Transplanted human cells populated both the myenteric and submucosal plexuses of the gut, demonstrating extensive migration within the gut wall and formation of neuronal networks with close interactions with the intact host ENS (Figure 4).

Transplantation studies using postnatal human and murine endogenous enteric neural stem cells in mice demonstrated functional integration (Cooper et al., 2016, 2017; Stamp et al., 2017), and rescue of an enteric neuropathy (McCann et al., 2017). Transplanted hPSC-derived ENS progenitors, generated through dual-SMAD inhibition, integrate and migrate extensively within a mouse model of HSCR leading to increased survival (Fattahi et al., 2016). Our work here extends and complements these studies providing further evidence to support the use of hPSCs as a promising platform for the development of cell therapies to treat ENS dysfunction.

## EXPERIMENTAL PROCEDURES

### hPSC Culture and Differentiation

The hESC lines H7 (WA07), H9 (WA09) (Thomson et al., 1998), H9*SOX10*:GFP (Chambers et al., 2012), clinical grade hESC line MasterShef7 and iPSC line SFCi55-ZsGr (Lopez-Yrigoyen et al., 2018) were maintained and NC differentiation performed as described previously (Frith et al., 2018). Enteric neurons were generated by plating day 10 spheres onto Geltrex-coated plates in BrainPhys (STEMCELL Technologies), supplemented with 1× N2, 1× B27, 100 μM ascorbic acid, 10 ng/mL GDNF, and 10 μM DAPT.Use of these Human ES cell lines for this project was approved by the UK Stem Cell Steering Committee, reference SCSC15-14. For full details, see Supplemental Information.

### RNA Extraction, cDNA Synthesis, and qPCR

Detailed methods and primer sequences can be found in the Supplemental Information.

### Immunofluorescence, Image Analysis, and Flow Cytometry

Detailed methods and materials can be found in the Supplemental Information.

### Animals

Animals were maintained, and experiments performed, in accordance with the UK Animals (Scientific Procedures) Act 1986 under license from the Home Office (P0336FFB0) and approved by the University College London Biological Services Ethical Review Process. Animal husbandry at UCL Biological Services was in accordance with the UK Home Office Certificate of Designation.

### *In vivo* Cell Transplantation

Day 6 sorted ZsGreen+/p75++ sorted vagal NC cells were plated into non-adherent plates in N2B27 medium supplemented with 3 μM CHIR99021, 20 ng/mL FGF2, and 10 μM Y27632-dihydrochloride. On day 7, cells were transplanted to the cecum of 4- to 8-week-old immunodeficient Rag2$^{-/-}$;γc$^{-/-}$;C5$^{-/-}$ mice via laparotomy under isofluorane anesthetic. Detailed methods are in the Supplemental Information.

### Whole Mount Gut Immunohistochemistry

Whole mount immunohistochemistry was performed on transplanted cecal and proximal colon segments after cervical dislocation and excision as per McCann et al. (2017). Detailed methods can be found in the Supplemental Information.

## SUPPLEMENTAL INFORMATION

Supplemental Information can be found online at https://doi.org/10.1016/j.stemcr.2020.07.024.

## AUTHOR CONTRIBUTIONS

T.J.R.F., P.W.A., J.O.S.H., C.J.McC., A.J.B., and N.T. conceived the project. T.J.R.F. and C.J.McC. designed, performed, and analyzed the experiments with help from A.G. Z.A.H. and H.D.M. derived the MasterShef7 hESC line. P.W.A., A.J.B., N.T., I.B., A.T., and C.J.McC. provided financial support. T.J.R.F., A.T., C.J.McC., and P.W.A. wrote the manuscript.

## ACKNOWLEDGMENTS

This project was supported by grants from the Medical Research Council Confidence in Concept awarded to I.B. and P.W.A. (MC_PC_14115), BBSRC (BB/P000444/1) awarded to A.T., and funding received from the European Union's Horizon 2020 Research and Innovation program H2020-FETPROACT-2018-01 under grant agreement no. 824070. C.J.McC. is supported by Guts UK (Derek Butler Fellowship). N.T. is supported by Great Ormond Street Hospital Children's Charity (GOSHCC - V1258). This

(D) Images of differentiated hPSC-derived ENS progenitors into nNOS+ and vAChT+ neurons in the cecum of Rag2$^{-/-}$;γc$^{-/-}$;C5$^{-/-}$ mice 3 months post-transplantation. Arrows show transplanted ZsGreen+ cells; arrowheads show endogenous enteric neurons. Scale bar = 50 μm.
(E) Table showing the numbers of mice in which ZsGreen+ cells were identified over the total number of transplanted mice analyzed at indicated time points post-transplantation.

work was partially funded by a GOSHCC grant (W1018C) to N.T. (Principal Investigator) and A.J.B. (Co-Investigator). We thank Lesley Forrester and Lorenz Studer for sharing the SFCi55-ZsGr iPSC and H9*SOX10*:GFP hESC lines, respectively. We acknowledge the NIHR Great Ormond Street Hospital Biomedical Research Center which supports all research at Great Ormond Street Hospital NHS Foundation Trust and UCL Great Ormond Street Institute of Child Health. The views expressed are those of the authors and not necessarily those of the NHS, the NIHR, or the Department of Health. We also acknowledge the support of Prince Abdullah Ben Khalid Celiac Research Chair, College of Medicine, Vice-Deanship of the Research Chairs, King Saud University, Riyadh, Saudi Arabia.

Received: October 25, 2019
Revised: July 28, 2020
Accepted: July 29, 2020
Published: August 27, 2020

## REFERENCES

Abu-Bonsrah, K.D., Zhang, D., Bjorksten, A.R., Dottori, M., and Newgreen, D.F. (2018). Generation of adrenal chromaffin-like cells from human pluripotent stem cells. Stem Cell Reports *10*, 134–150.

Baynash, A.G., Hosoda, K., Giaid, A., Richardson, J.A., Emoto, N., Hammer, R.E., and Yanagisawa, M. (1994). Interaction of endothelin-3 with endothelin-B receptor is essential for development of epidermal melanocytes and enteric neurons. Cell *79*, 1277–1285.

Blaugrund, E., Pham, T.D., Tennyson, V.M., Lo, L., Sommer, L., Anderson, D.J., and Gershon, M.D. (1996). Distinct subpopulations of enteric neuronal progenitors defined by time of development, sympathoadrenal lineage markers and Mash-1-dependence. Development *122*, 309–320.

Bondurand, N., Natarajan, D., Barlow, A., Thapar, N., and Pachnis, V. (2006). Maintenance of mammalian enteric nervous system progenitors by SOX10 and endothelin 3 signalling. Development *133*, 2075–2086.

Chambers, S.M., Qi, Y., Mica, Y., Lee, G., Zhang, X.-J., Niu, L., Bilsland, J., Cao, L., Stevens, E., Whiting, P., et al. (2012). Combined small-molecule inhibition accelerates developmental timing and converts human pluripotent stem cells into nociceptors. Nat. Biotechnol. *30*, 715–720.

Chan, K.K., Chen, Y.S., Yau, T.O., Fu, M., Lui, V.C.H., Tam, P.K.H., and Sham, M.H. (2005). Hoxb3 vagal neural crest-specific enhancer element for controlling enteric nervous system development. Dev. Dyn. *233*, 473–483.

Cooper, J.E., McCann, C.J., Natarajan, D., Choudhury, S., Boesmans, W., Delalande, J.-M., Vanden Berghe, P., Burns, A.J., and Thapar, N. (2016). In vivo transplantation of enteric neural crest cells into mouse gut; engraftment, functional integration and long-term safety. PLoS One *11*, e0147989.

Cooper, J.E., Natarajan, D., McCann, C.J., Choudhury, S., Godwin, H., Burns, A.J., and Thapar, N. (2017). In vivo transplantation of fetal human gut-derived enteric neural crest cells. Neurogastroenterol. Motil. *29*, e12900.

Diman, N.Y.S.-G., Remacle, S., Bertrand, N., Picard, J.J., Zaffran, S., and Rezsohazy, R. (2011). A retinoic acid responsive Hoxa3 transgene expressed in embryonic pharyngeal endoderm, cardiac neural crest and a subdomain of the second heart field. PLoS One *6*, e27624.

Durbec, P., Marcos-Gutierrez, C.V., Kilkenny, C., Grigoriou, M., Wartiowaara, K., Suvanto, P., Smith, D., Ponder, B., Costantini, F., Saarma, M., et al. (1996). GDNF signalling through the Ret receptor tyrosine kinase. Nature *381*, 789–793.

El Robrini, N., Etchevers, H.C., Ryckebusch, L., Faure, E., Eudes, N., Niederreither, K., Zaffran, S., and Bertrand, N. (2016). Cardiac outflow morphogenesis depends on effects of retinoic acid signaling on multiple cell lineages. Dev. Dyn. *245*, 388–401.

Elworthy, S., Pinto, J.P., Pettifer, A., Cancela, M.L., and Kelsh, R.N. (2005). Phox2b function in the enteric nervous system is conserved in zebrafish and is sox10-dependent. Mech. Dev. *122*, 659–669.

Fattahi, F., Steinbeck, J.A., Kriks, S., Tchieu, J., Zimmer, B., Kishinevsky, S., Zeltner, N., Mica, Y., El-Nachef, W., Zhao, H., et al. (2016). Deriving human ENS lineages for cell therapy and drug discovery in Hirschsprung disease. Nature *531*, 105–109.

Frith, T.J., Granata, I., Wind, M., Stout, E., Thompson, O., Neumann, K., Stavish, D., Heath, P.R., Ortmann, D., Hackland, J.O., et al. (2018). Human axial progenitors generate trunk neural crest cells in vitro. eLife *7*, 134.

Fu, M., Lui, V.C.H., Sham, M.H., Cheung, A.N.Y., and Tam, P.K.H. (2003). HOXB5 expression is spatially and temporarily regulated in human embryonic gut during neural crest cell colonization and differentiation of enteric neuroblasts. Dev. Dyn. *228*, 1–10.

Hackland, J.O.S., Frith, T.J.R., Thompson, O., Marin Navarro, A., García-Castro, M.I., Unger, C., and Andrews, P.W. (2017). Top-down inhibition of BMP signaling enables robust induction of hPSCs into neural crest in fully defined, xeno-free conditions. Stem Cell Reports *9*, 1043–1052.

Hackland, J.O.S., Shelar, P.B., Sandhu, N., Prasad, M.S., Charney, R.M., Gomez, G.A., Frith, T.J.R., and García-Castro, M.I. (2019). FGF modulates the axial identity of trunk hPSC-derived neural crest but not the cranial-trunk decision. Stem Cell Reports *12*, 920–933.

Hosoda, K., Hammer, R.E., Richardson, J.A., Baynash, A.G., Cheung, J.C., Giaid, A., and Yanagisawa, M. (1994). Targeted and natural (piebald-lethal) mutations of endothelin-B receptor gene produce megacolon associated with spotted coat color in mice. Cell *79*, 1267–1276.

Hutchins, E.J., Kunttas, E., Piacentino, M.L., Howard, A.G.A., Bronner, M.E., and Uribe, R.A. (2018). Migration and diversification of the vagal neural crest. Dev. Biol. *444*, S98–S109.

Ishikawa, S., and Ito, K. (2009). Plasticity and regulatory mechanisms of Hox gene expression in mouse neural crest cells. Cell Tissue Res. *337*, 381–391.

Kam, M.K.M., and Lui, V.C.H. (2015). Roles of Hoxb5 in the development of vagal and trunk neural crest cells. Dev. Growth Differ. *57*, 158–168.

Kam, M.K.M., Cheung, M.C.H., Zhu, J.J., Cheng, W.W.C., Sat, E.W.Y., Tam, P.K.H., and Lui, V.C.H. (2014). Perturbation of

Hoxb5 signaling in vagal and trunk neural crest cells causes apoptosis and neurocristopathies in mice. Cell Death Differ. *21*, 278–289.

Lai, F.P.-L., Lau, S.-T., Wong, J.K.-L., Gui, H., Wang, R.X., Zhou, T., Lai, W.H., Tse, H.-F., Tam, P.K.H., Garcia-Barcelo, M.M., and Ngan, E.S.-W. (2017). Correction of Hirschsprung-associated mutations in human induced pluripotent stem cells via clustered regularly interspaced short palindromic repeats/Cas9, restores neural crest cell function. Gastroenterology *153*, 139–153.

Lasrado, R., Boesmans, W., Kleinjung, J., Pin, C., Bell, D., Bhaw, L., McCallum, S., Zong, H., Luo, L., Clevers, H., et al. (2017). Lineage-dependent spatial and functional organization of the mammalian enteric nervous system. Science *356*, 722–726.

Le Douarin, N.M., Creuzet, S., Couly, G., and Dupin, E. (2004). Neural crest cell plasticity and its limits. Development *131*, 4637–4650.

Li, W., Huang, L., Zeng, J., Lin, W., Li, K., Sun, J., Huang, W., Chen, J., Wang, G., Ke, Q., et al. (2016). Characterization and transplantation of enteric neural crest cells from human induced pluripotent stem cells. Mol. Psychiatry *2018*, 499–508.

Lippmann, E.S., Williams, C.E., Ruhl, D.A., Estevez-Silva, M.C., Chapman, E.R., Coon, J.J., and Ashton, R.S. (2015). Deterministic HOX patterning in human pluripotent stem cell-derived neuroectoderm. Stem Cell Reports *4*, 632–644.

Lo, L.C., Johnson, J.E., Wuenschell, C.W., Saito, T., and Anderson, D.J. (1991). Mammalian achaete-scute homolog 1 is transiently expressed by spatially restricted subsets of early neuroepithelial and neural crest cells. Genes Dev. *5*, 1524–1537.

Lopez-Yrigoyen, M., Fidanza, A., Cassetta, L., Axton, R.A., Taylor, A.H., Meseguer-Ripolles, J., Tsakiridis, A., Wilson, V., Hay, D.C., Pollard, J.W., and Forrester, L.M. (2018). A human iPSC line capable of differentiating into functional macrophages expressing ZsGreen: a tool for the study and in vivo tracking of therapeutic cells. Philos. Trans. R. Soc. Lond. Ser. B Biol. Sci. *373*, 20170219.

McCann, C.J., Cooper, J.E., Natarajan, D., Jevans, B., Burnett, L.E., Burns, A.J., and Thapar, N. (2017). Transplantation of enteric nervous system stem cells rescues nitric oxide synthase deficient mouse colon. Nat. Commun. *8*, 15937.

Memic, F., Knoflach, V., Sadler, R., Tegerstedt, G., Sundström, E., Guillemot, F., Pachnis, V., and Marklund, U. (2016). Ascl1 is required for the development of specific neuronal subtypes in the enteric nervous system. J. Neurosci. *36*, 4339–4350.

Niederreither, K., Vermot, J., Le Roux, I., Schuhbaur, B., Chambon, P., and Dollé, P. (2003). The regional pattern of retinoic acid synthesis by RALDH2 is essential for the development of posterior pharyngeal arches and the enteric nervous system. Development *130*, 2525–2534.

Niederreither, K., Vermot, J., Messaddeq, N., Schuhbaur, B., Chambon, P., and Dolle, P. (2001). Embryonic retinoic acid synthesis is essential for heart morphogenesis in the mouse. Development *128*, 1019–1031.

Okada, Y., Shimazaki, T., Sobue, G., and Okano, H. (2004). Retinoic-acid-concentration-dependent acquisition of neural cell identity during in vitro differentiation of mouse embryonic stem cells. Dev. Biol. *275*, 124–142.

Okamura, Y., and Saga, Y. (2008). Notch signaling is required for the maintenance of enteric neural crest progenitors. Development *135*, 3555–3565.

Papalopulu, N., Clarke, J.D., Bradley, L., Wilkinson, D., Krumlauf, R., and Holder, N. (1991). Retinoic acid causes abnormal development and segmental patterning of the anterior hindbrain in *Xenopus* embryos. Development *113*, 1145–1158.

Sasselli, V., Pachnis, V., and Burns, A.J. (2012). The enteric nervous system. Dev. Biol. *366*, 64–73.

Simeone, A., Acampora, D., Arcioni, L., Andrews, P.W., Boncinelli, E., and Mavilio, F. (1990). Sequential activation of HOX2 homeobox genes by retinoic acid in human embryonal carcinoma cells. Nature *346*, 763–766.

Simkin, J.E., Zhang, D., Rollo, B.N., and Newgreen, D.F. (2013). Retinoic acid upregulates ret and induces chain migration and population expansion in vagal neural crest cells to colonise the embryonic gut. PLoS One *8*, e64077.

Simkin, J.E., Zhang, D., Stamp, L.A., and Newgreen, D.F. (2018). Fine scale differences within the vagal neural crest for enteric nervous system formation. Dev. Biol. *446*, 22–33.

Stamp, L.A., Gwynne, R.M., Foong, J.P.P., Lomax, A.E., Hao, M.M., Kaplan, D.I., Reid, C.A., Petrou, S., Allen, A.M., Bornstein, J.C., and Young, H.M. (2017). Optogenetic demonstration of functional innervation of mouse colon by neurons derived from transplanted neural cells. Gastroenterology *152*, 1407–1418.

Stuhlmiller, T.J., and García-Castro, M.I. (2012). Current perspectives of the signaling pathways directing neural crest induction. Cell Mol. Life Sci. *69*, 3715–3737.

Theocharatos, S., Wilkinson, D.J., Darling, S., Wilm, B., Kenny, S.E., and Edgar, D. (2013). Regulation of progenitor cell proliferation and neuronal differentiation in enteric nervous system neurospheres. PLoS One *8*, e54809.

Thomson, J.A., Itskovitz-Eldor, J., Shapiro, S.S., Waknitz, M.A., Swiergiel, J.J., Marshall, V.S., and Jones, J.M. (1998). Embryonic stem cell lines derived from human blastocysts. Science *282*, 1145–1147.

Uribe, R.A., and Bronner, M.E. (2015). Meis3 is required for neural crest invasion of the gut during zebrafish enteric nervous system development. Mol. Biol. Cell *26*, 3728–3740.

Uribe, R.A., Hong, S.S., and Bronner, M.E. (2018). Retinoic acid temporally orchestrates colonization of the gut by vagal neural crest cells. Dev. Biol. *433*, 17–32.

Workman, M.J., Mahe, M.M., Trisno, S., Poling, H.M., Watson, C.L., Sundaram, N., Chang, C.-F., Schiesser, J., Aubert, P., Stanley, E.G., et al. (2016). Engineered human pluripotent-stem-cell-derived intestinal tissues with a functional enteric nervous system. Nat. Med. *23*, 49–59.

Zhu, J.J., Kam, M.K., Garcia-Barceló, M.-M., Tam, P.K.H., and Lui, V.C.H. (2014). HOXB5 binds to multi-species conserved sequence (MCS+9.7) of RET gene and regulates RET expression. Int. J. Biochem. Cell Biol. *51*, 142–149.

Stem Cell Reports, Volume *15*

## Supplemental Information

# Retinoic Acid Accelerates the Specification of Enteric Neural Progenitors from *In-Vitro*-Derived Neural Crest

**Thomas J.R. Frith, Antigoni Gogolou, James O.S. Hackland, Zoe A. Hewitt, Harry D. Moore, Ivana Barbaric, Nikhil Thapar, Alan J. Burns, Peter W. Andrews, Anestis Tsakiridis, and Conor J. McCann**

# FIGURE S1

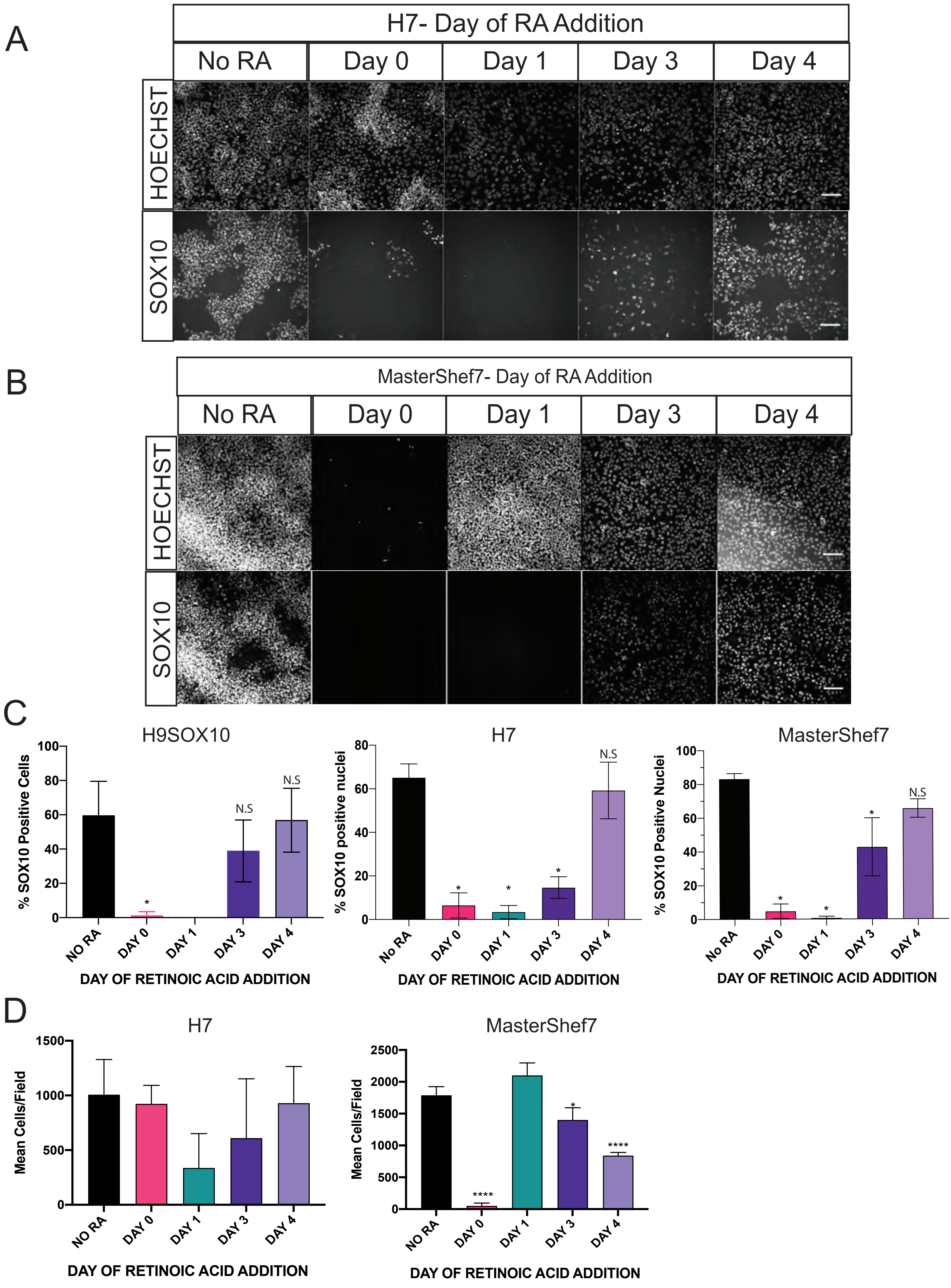
A
H7- Day of RA Addition
No RA
Day 0
Day 1
Day 3
Day 4
HOECHST
SOX10
B
MasterShef7- Day of RA Addition
No RA
Day 0
Day 1
Day 3
Day 4
HOECHST
SOX10
C
H9SOX10
% SOX10 Positive Cells
100
80
60
40
20
0
N.S
N.S
*
NO RA
DAY 0
DAY 1
DAY 3
DAY 4
DAY OF RETINOIC ACID ADDITION
H7
% SOX10 positive nuclei
80
60
40
20
0
N.S
*
*
*
No RA
DAY 0
DAY 1
DAY 3
DAY 4
DAY OF RETINOIC ACID ADDITION
MasterShef7
% SOX10 Positive Nuclei
100
80
60
40
20
0
N.S
*
*
*
No RA
DAY 0
DAY 1
DAY 3
DAY 4
DAY OF RETINOIC ACID ADDITION
D
H7
Mean Cells/Field
1500
1000
500
0
NO RA
DAY 0
DAY 1
DAY 3
DAY 4
DAY OF RETINOIC ACID ADDITION
MasterShef7
Mean Cells/Field
2500
2000
1500
1000
500
0
****
*
****
NO RA
DAY 0
DAY 1
DAY 3
DAY 4
DAY OF RETINOIC ACID ADDITION

# FIGURE S2

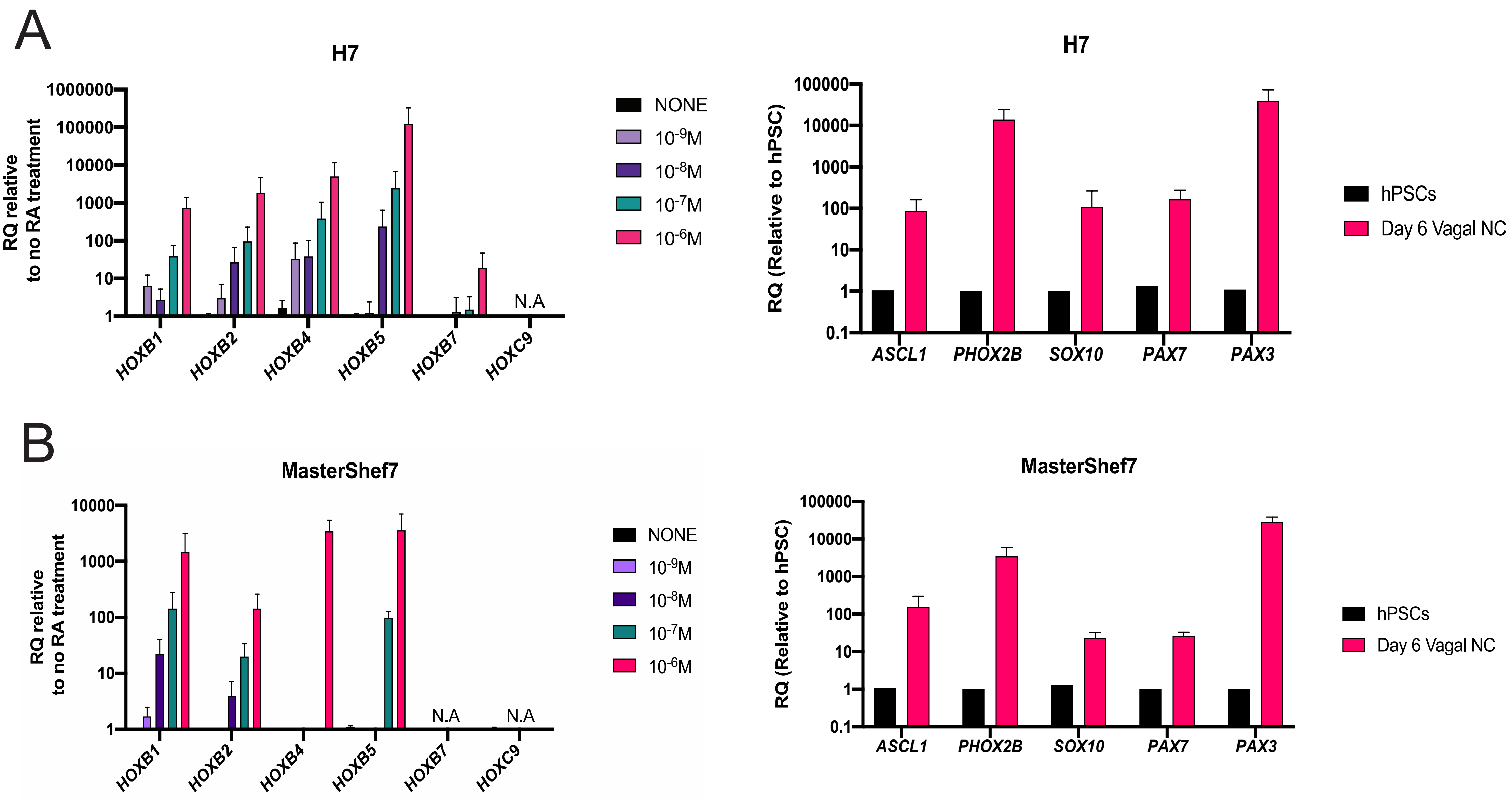
A
H7
RQ relative to no RA treatment
1000000
100000
10000
1000
100
10
1
HOXB1
HOXB2
HOXB4
HOXB5
HOXB7
HOXC9
N.A
NONE
10-9M
10-8M
10-7M
10-6M
H7
RQ (Relative to hPSC)
100000
10000
1000
100
10
1
0.1
ASCL1
PHOX2B
SOX10
PAX7
PAX3
hPSCs
Day 6 Vagal NC
B
MasterShef7
RQ relative to no RA treatment
10000
1000
100
10
1
HOXB1
HOXB2
HOXB4
HOXB5
HOXB7
HOXC9
N.A
N.A
NONE
10-9M
10-8M
10-7M
10-6M
MasterShef7
RQ (Relative to hPSC)
100000
10000
1000
100
10
1
0.1
ASCL1
PHOX2B
SOX10
PAX7
PAX3
hPSCs
Day 6 Vagal NC

# FIGURE S3

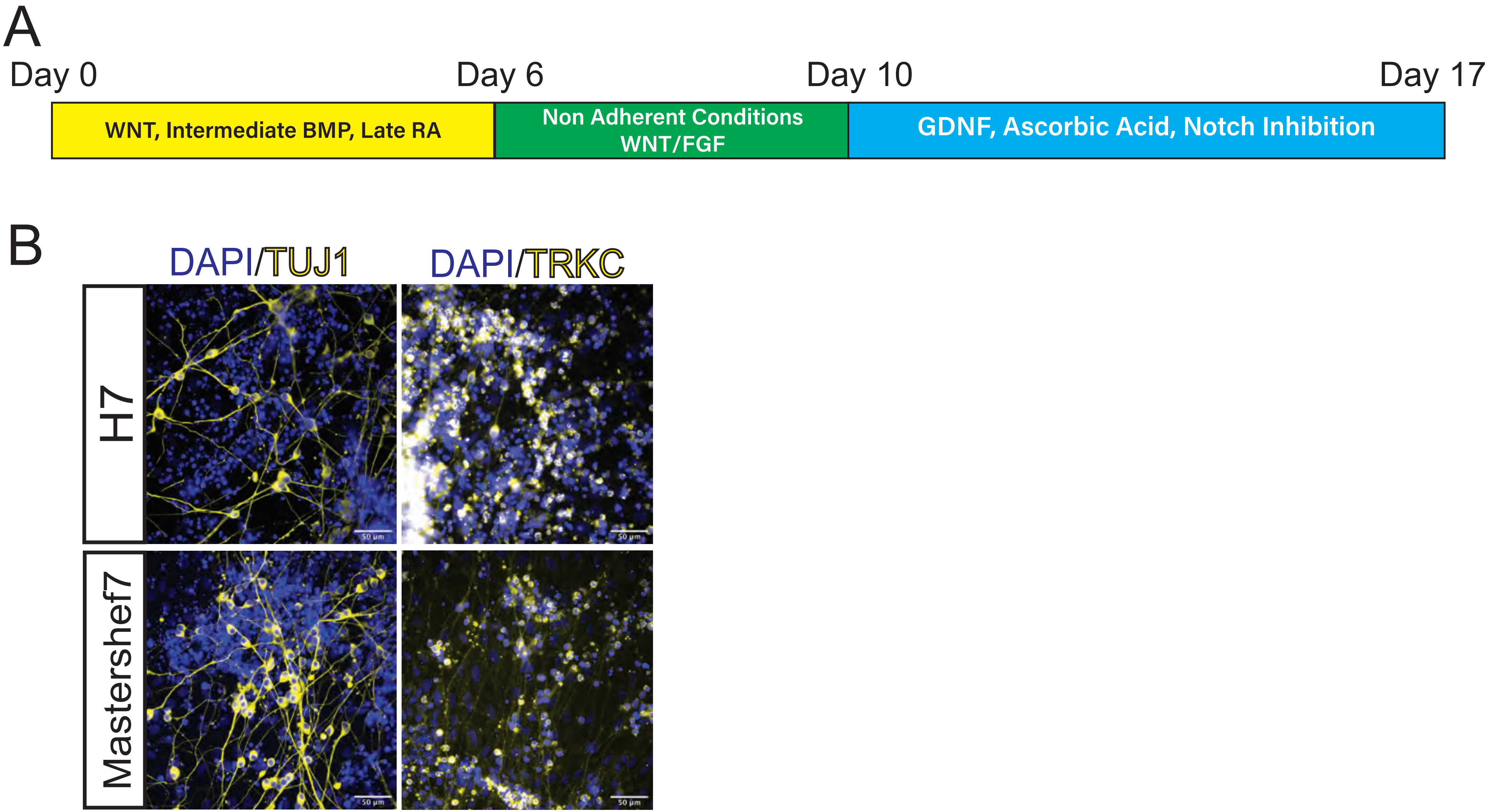
A
Day 0
Day 6
Day 10
Day 17
WNT, Intermediate BMP, Late RA
Non Adherent Conditions
WNT/FGF
GDNF, Ascorbic Acid, Notch Inhibition
B
DAPI/TUJ1
DAPI/TRKC
H7
Mastershef7

**Supplementary Figure legends**

**Supplementary Figure 1: RA timing conserved across other hPSC lines.**

**(A-B)** Immunofluorescence images showing SOX10 expression at day 5 after addition of RA at different times during NC differentiation of H7 **(A)** and MasterShef7 **(B)** hPSCs. Scale Bar = 100µm

**(C)** Quantification of *SOX10*:GFP positive (4 biological repeats) and SOX10 positive cells in H7 (N=3 biological repeats) and MasterShef7 (N=3 biological repeats) after addition of RA at different time points. Bars=mean; error=s.d. *P<0.05, N.S= not significant. One-way ANOVA of cells treated with RA compared to day 5 cells not treated with RA.

**(D)** Count of the mean number of positive HOECHST nuclei per field for H7 and MasterShef7 following the addition of RA at different timepoints during neural crest differentiation. N=3 independent differentiations. Bars=mean; error=s.d; One-way ANOVA * P<0.05, **** P<0.0001

**Supplementary Figure 2: HOX gene induction and early enteric neural marker induction is dependent on the concentration of RA**

qPCR analysis showing *HOX* gene and early ENS progenitor marker induction after 6 days of differentiation following RA exposure of **(A)** H7 and **(B)** MasterShef7 hPSCs. N.A = no amplification. N= 3 independent differentiations per line

**Supplementary Figure 3: Generation of enteric neurons in 2 further independent hPSC lines.**

**(A)** Enteric neuron differentiation protocol.

**(B)** Immunofluorescence showing the cells that are positive for TUJ1 and TRKC at day 17 of differentiation. Scale = 50µm. N=3 independent differentiations.

## Supplemental Methods

### hPSC Culture

The hESC lines H7 (WA07), H9 (WA09) (Thomson et al., 1998), H9:SOX10 (Chambers et al., 2012), clinical grade hESC line MasterShef7 and iPSC line SFCi55-ZsGr (Lopez-Yrigoyen et al., 2018) were grown in mTESR (Stem Cell Technologies # 85850) on 1:100 dilution of Geltrex (ThermoFisher A1413202) in DMEM/F12 (Sigma D6421). Cells were passaged at 80-90% confluency using ReLeSR (Stem Cell Technologies Catalog # 05873). Cells were incubated at 37°C in 5%$CO_2$. Use of these Human ES cell lines for this project was approved by the UK Stem Cell Steering Committee, reference SCSC15-14.

The clinical grade hESC line, MasterShef7 (https://hpscreg.eu/cell-line/UOSe012-A) was derived at the University of Sheffield, Centre for Stem Cell Biology, under GMP-like conditions in a cleanroom setting (HFEA licence R115-8-A (Centre 0191) and HTA licence 22510).

The frozen embryo was surplus for IVF treatment, donated with fully informed consent, with no financial benefit to the donors and cultured to the blastocyst stage using IVF media (Medicult). Following removal of the trophectoderm using a

dissection laser the embryo was explanted whole onto mitotically inactivated human neonatal fibroblasts (human feeders) in standard KSR/KODMEM (Life Technologies) medium.

MasterShef7 was initially maintained at 37°C under 5% O2 /5% CO2, until established, after which maintenance switched 5% CO2 in air at 37°C. Cultures were passaged using a manual technique, cutting selected colonies under a dissection microscope at an average split ratio of 1:2 every 7 days.
MasterShef7 has been deposited at the UK Stem Cell bank (https://www.nibsc.org/ukstemcellbank) in line with the requirements of the HFEA licence.

**Directed Differentiation**

For vagal neural crest differentiation, we use a previously described protocol (Frith et al., 2018). hPSCs at approximately 80% confluency were detached using Accutase (Sigma-Aldrich A6964) for 10 minutes at 37°C to generate single cells. Cells were counted manually and plated at 50,000 cells/$cm^2$ on Geltrex coated plates (ThermoFisher A1413202). Neural Crest differentiation media is comprised of DMEM/F12 (Sigma-Aldrich), supplemented with 1x N2 (ThermoFisher 17502048), NEAA (ThermoFisher 11140050), Glutamax (ThermoFisher 35050061), 1µM CHIR99021 (Tocris 4423), 2µM SB431542 (Tocris 1614/1), 1µM DMH-1 (Tocris 4126/10), 20ng/ml BMP4 (ThermoFisher PHC9533). All-Trans Retinoic Acid (Sigma R2625) was diluted in DMSO. 10µM Y-27632 dihydrochloride (Tocris 1254/1) was added at day 0 until day 2 to assist attachment. For all vagal neural crest induction all-trans Retinoic acid was added at a final concentration of 1µM on day 4 unless specified in the results. Media was changed every other day until day 5/6.

**Sphere Formation**

Spheres were generated as previously described (Fattahi et al., 2016). Day 6 cells were treated with accutase to form a single cell suspension and replated in a media containing a 1:1 mix of DMEM/F12 (Sigma) with Neurobasal (ThermoFisher 21103049) supplemented with 1x N2, 1x B27, 1x NEAA, 1x Glutamax, 3µM CHIR99021, 10ng/ml FGF2 (R&D systems 233-FB/CF). Sphere media supplemented 10µM of Y-27632 dihydrochloride (Tocris) to ensure sphere formation and left until day 10. One well of a 6 well plate was plated into one well of an Ultra-Low Attachment 6 well plate (Corning 3471) or 6 well plates with a coating of 1% w/v agarose.

**Enteric Neuron Differentiation**

For enteric neuronal differentiation, day 10 spheres were plated onto Geltrex coated plates in BrainPhys (Stem Cell Technologies 05790) supplemented with 1x N2, 1x B27 (ThermoFisher 17504044), 100µM Ascorbic Acid (Sigma A8960), 10ng/ml GDNF (Peprotech 450-10) and 10µM DAPT (Sigma D5942). Media was changed every other day and once a week supplemented with Vitronectin (ThermoFisher A14700)

**Flow Cytometry**

A single cell suspension was generated using Accutase as described above. Cells were pelleted and resuspended in FACS buffer (DMEM/10% v/v FCS) at $1x10^6$ cells/ml. Gating for positive cells was based on a negative control consisting of cells not carrying a reporter or cells stained with P3X, an antibody from the parent myeloma (KOHLER and MILSTEIN, 1975; Hackland et al., 2017).

**RNA extraction, cDNA synthesis & qPCR**

RNA was extracted using a Total RNA purification plus kit (Norgen BioTek #48300) per manufacturer's instructions. RNA concentration was measured using a nanodrop (ThermoFisher). RNA was stored at -80° C. cDNA was synthesised using the High-Capacity cDNA Reverse Transcription kit (ThermoFisher 4368813) and stored at -20°C.

qPCR was performed on QuantStudio 12K Flex thermocycler (Applied Biosystems). CT values were calculated against GAPDH for each sample. Relative quantities calculated using the -2^^CT method. Primer sequences can be found in Tables 2 & 3.

**Immunofluorescence & Image Analysis**

Cells were fixed with 4% PFA for 10 minutes at room temperature and washed 3 times with 1x PBS (no $Mg^{2+}$/ $Ca^{2+}$). Cells were permeabilised and blocked with 1x PBS (no $Mg^{2+}$/ $Ca^{2+}$) supplemented with 10% FCS, 0.1% BSA and 0.3% Triton-X 100 for 1 hour at room temperature. Primary antibodies were diluted in permeabilisation buffer and incubated at 4°C overnight. Secondary antibodies were diluted in permeabilization buffer and stained in the dark at 4°C for 1 hour. Nuclei were counterstained with Hoechst 33342 (ThermoFisher H3570). Images were taken on an InCell Analyser 2500 (GE Healthcare) and quantified using custom made pipelines on CellProfiler 2.2 (Carpenter et al., 2006) as per (Frith et al., 2018).

**Table 1: Antibodies used in the study**

| | Antibody | Species | Source | Dilution |
|---|---|---|---|---|
| In Vitro | SOX10 | Rabbit | Cell Signalling Technology (D5V9L) #89356 | 1:500 |
| | RET | Rabbit | Abcam ab134100 | 1:1000 |
| | TUJ1 | Mouse | Abcam ab78078 | 1:1000 |
| | TRKC | Rabbit | Cell Signalling Technology (C44H5) #3376 | 1:1000 |
| | PERIPHERIN | Rabbit | Millipore AB1530 | 1:100 |
| | P3X | Mouse | In house myeloma P3X63Ag8 (Kohler & Milstein 1975) | 1:10 |
| | p75 | Mouse | In house hybridoma Clone ME20.4 (Ross et al., 1984) | 1:20 |
| | CD49d | Mouse | BioLegend 304302 Clone 9F10 | 1:100 |
| Transplants | TUJ1 | Mouse | BioLegend MMS-435P | 1:500 |
| | GFAP | Rabbit | Millipore AB5804 | 1:500 |
| | nNOS | Rabbit | Invitrogen 61-7000 | 1:400 |
| | vAChT | Goat | ThermoFisher Scientific OSH00003W | 1:200 |
| | DAPI | | Sigma D8417 | 1:1000 |

**Table 2: Primers and UPL probes used in study**

| Gene | Forward | Reverse | Roche UPL Probe |
|---|---|---|---|
| *GAPDH* | agccacatcgctcagacac | gcccaatacgaccaaatcc | 60 |
| *HOXB1* | ccagctagggggcttgtc | atgctgcggaggatatgg | 39 |
| *HOXB2* | aatccgccacgtctcctt | gctgcgtgttggtgtaagc | 70 |
| *HOXB4* | ctggatgcgcaaagttcac | agcggttgtagtgaaattcctt | 62 |
| *HOXB5* | aagcttcacatcagccatga | cggttgaagtggaactccttt | 1 |
| *HOXB7* | ctacccctggatgcgaag | caggtagcgattgtagtgaaattct | 1 |
| *HOXC9* | gcagcaagcacaaagagga | cgtctggtacttggtgtaggg | 85 |
| *SOX10* | ggctcccccatgtcagat | ctgtcttcggggtggttg | 21 |
| *PAX3* | aggaggccgacttggaga | cttcatctgattggggtgct | 13 |
| *PAX7* | gaaaacccaggcatgttcag | gcggctaatcgaactcactaa | 66 |
| *ASCL1* | cgacttcaccaactggttctg | atgcaggttgtgcgatca | 38 |
| *PHOX2A* | cactaccccgacatttacacg | gctcctgtttgcggaactt | 17 |
| *PHOX2B* | ctaccccgacatctacactcg | ctcctgcttgcgaaacttg | 17 |
| *SST* | accccagactccgtcagttt | acagcagctctgccaagaag | 38 |
| *CHAT* | cagccctgatgccttcat | cagtcttcgatggagcctgt | 78 |
| *TH* | acgccaaggacaagctca | agcgtgtacgggtcgaact | 42 |
| *HTR2a* | tgatgtcacttgccatagctg | caggtaaatccagactgcacaa | 3 |
| *GFRA1* | caccattgccctgaaagaat | cgcttttaggggttcaggtc | 36 |

**Table 3: Taqman Gene Expression Assays for Figure 3G. Thermo Fisher Scientific Cat # 4331182**

| Gene | Assay ID |
|---|---|
| *GAPDH* | Hs03929097_g1 |
| *PLP1* | Hs00166914_m1 |
| *S100β* | Hs00902901_m1 |
| *FABP7* | Hs00361424_g1 |
| *ERBB3* | Hs00176538_m1 |
| *SOX10* | Hs00366918_m1 |

**In vivo cell transplantation**

Day 6 P75++/ZsGreen+ cells were purified and grown as spheres for one day as outlined above and transplanted the following day (day 7 of differentiation). Briefly, the caecum was exposed and ZsGreen+ spheres, containing 1 million cells each, were subsequently transplanted to the serosal aspect of the caecum by mouth pipette, using a pulled glass micropipette. Each transplanted tissue typically received 3 ZsGreen+ spheres which were manipulated on the surface of the caecum with the bevel of a 30G needle to ensure correct positioning. Transplanted $Rag2^{-/-};\gamma c^{-/-};C5^{-/-}$mice were typically maintained for either 2 weeks (N=2), 4 weeks (N=9) or 3 months (N=4) post-transplantation, before sacrifice and removal of the caecum and proximal colon for analysis.

**Animals**

$Rag2^{-/-};\gamma c^{-/-};C5^{-/-}$ mice, which lack innate immunity, and are deficient in all lymphocytes (Cooper et al., 2003; Silva-Barbosa et al., 2005), were used as recipients for all transplants.

**Wholemount Gut Immunohistochemistry**

Tissues were fixed in ice cold 4% PFA for 45 min at 22°C. After fixation, tissues were washed for 24h in 1x PBS at 4°C. Cells were permeabilised and blocked with 1x PBS supplemented with 1% Triton X-100 and 10% sheep serum. Primary antibodies were diluted in permeabilisation buffer and incubated at 4°C for 48h. Secondary antibodies were diluted in permeabilisation buffer and stained in the dark for one hour at 22°C. Nuclei were counterstained with DAPI (Sigma). Before mounting, tissues were washed thoroughly in 1x PBS for 2h at 22 °C. Tissues were examined using a LSM710 Meta confocal microscope (Zeiss). Confocal micrographs of whole mounts were digital composites of the Z-series of scans (0.5-1μm optical sections, 10–50μm thick).

**Supplemental References**


Carpenter, A.E., Jones, T.R., Lamprecht, M.R., Clarke, C., Kang, I.H., Friman, O., Guertin, D.A., Chang, J.H., Lindquist, R.A., Moffat, J., Golland, P. and Sabatini, D.M. 2006. CellProfiler: image analysis software for identifying and quantifying cell phenotypes. *Genome biology*. **7**. R100.

Chambers, S.M., Qi, Y., Mica, Y., Lee, G., Zhang, X.-J., Niu, L., Bilsland, J., Cao, L., Stevens, E., Whiting, P., Shi, S.-H. and Studer, L. 2012. Combined small-molecule inhibition accelerates developmental timing and converts human pluripotent stem cells into nociceptors. *Nat Biotechnol*. **30**. 715–720.

Cooper, R.N., Thiesson, D., Furling, D., Di Santo, J.P., Butler-Browne, G.S. and Mouly, V. 2003. Extended amplification in vitro and replicative senescence: key factors implicated in the success of human myoblast transplantation. *Human gene therapy*. **14**. 1169–1179.

Fattahi, F., Steinbeck, J.A., Kriks, S., Tchieu, J., Zimmer, B., Kishinevsky, S., Zeltner, N., Mica, Y., El-Nachef, W., Zhao, H., de Stanchina, E., Gershon, M.D., Grikscheit, T.C., Chen, S. and Studer, L. 2016. Deriving human ENS lineages for cell therapy and drug discovery in Hirschsprung disease. *Nature*. **531**.105–109.

Frith, T.J., Granata, I., Wind, M., Stout, E., Thompson, O., Neumann, K., Stavish, D., Heath, P.R., Ortmann, D., Hackland, J.O., Anastassiadis, K., Gouti, M., Briscoe, J., Wilson, V., Johnson, S.L., Placzek, M., Guarracino, M.R., Andrews, P.W. and Tsakiridis, A. 2018. Human axial progenitors generate trunk neural crest cells in vitro. *Elife*. **7**. 134.

Hackland, J.O.S., Frith, T.J.R., Thompson, O., Marin Navarro, A., García-Castro, M.I., Unger, C. and Andrews, P.W. 2017. Top-Down Inhibition of BMP Signaling Enables Robust Induction of hPSCs Into Neural Crest in Fully Defined, Xeno-free Conditions. *Stem Cell Reports*. **9**. 1043–1052.

KOHLER, G. and MILSTEIN, C. 1975. Continuous cultures of fused cells secreting antibody of predefined specificity. *Nature*. **256**. 495–497.

Lopez-Yrigoyen, M., Fidanza, A., Cassetta, L., Axton, R.A., Taylor, A.H., Meseguer-Ripolles, J., Tsakiridis, A., Wilson, V., Hay, D.C., Pollard, J.W. and Forrester, L.M. 2018. A human iPSC line capable of differentiating into functional macrophages expressing ZsGreen: a tool for the study and in vivo tracking of therapeutic cells. *Philosophical transactions of the Royal Society of London. Series B, Biological sciences*. **373**(1750).

Ross, A.H., Grob, P., Bothwell, M., Elder, D.E., Ernst, C.S., Marano, N., Ghrist, B.F., Slemp, C.C., Herlyn, M. and Atkinson, B. 1984. Characterization of nerve growth factor receptor in neural crest tumors using monoclonal antibodies. *Proc Natl Acad Sci U S A*. **81**. 6681–6685.

Silva-Barbosa, S.D., Butler-Browne, G.S., Di Santo, J.P. and Mouly, V. 2005. Comparative analysis of genetically engineered immunodeficient mouse strains as recipients for human myoblast transplantation. *Cell transplantation*. **14**. 457–467.

Thomson, J.A, Itskovitz-Eldor, J., Shapiro, S.S, Waknitz, M. A., Swiergiel, J.J., Marshall, V. S., Jones, J. M., 1998. Embryonic Stem Cell Lines Derived from Human Blastocysts. *Science*. **282**. 1145–1147.
